# Study on Erosion Patterns of Cyclone Desanders at Shale Gas Wellheads

**DOI:** 10.3390/ma19102094

**Published:** 2026-05-16

**Authors:** Qian Huang, Chi Zhang, Peng Zou, Jingxi Hu, Zhitao Hou, Hao Jiao, Yuan Tian, Huirong Huang, Jiang Meng, Xueyuan Long

**Affiliations:** 1 College of Petroleum and Natural Gas Engineering, Chongqing University of Science and Technology, Chongqing 401331, China; huangqianswpu@163.com (Q.H.); 2025201122@cqust.edu.cn (J.H.); 2025201134@cqust.edu.cn (Z.H.); 2007079@cqust.edu.cn (Y.T.); hryh@cqust.edu.cn (H.H.); cqmj07@163.com (J.M.); 2 Construction Project Management Branch of National Oil and Gas Pipeline Network Group Co., Ltd., Chengdu 610200, China; 19115651919@163.com; 3 School of Safety Science and Engineering, Chongqing University of Science and Technology, Chongqing 401331, China; 2025208001@cqust.edu.cn (H.J.); 2007078@cqust.edu.cn (X.L.)

**Keywords:** cyclone desander, erosive wear, computational fluid dynamics, response surface methodology, predictive modeling

## Abstract

In shale gas extraction, solid particles such as fracturing proppants cause erosion in production and transmission pipelines. Cyclone desanders are widely used for gas–solid separation, but high-velocity sand-laden fluids frequently induce equipment failure, leakage and safety risks. Therefore, research on erosion and protective measures is essential. This study focuses on the desander at the M shale gas wellhead, where wall thickness was measured at three monitoring points to determine erosion rates. A CFD-based numerical erosion model for the cyclone desander was developed using ANSYS Fluent within the ANSYS Workbench 19.2 environment (ANSYS, Inc., Canonsburg, PA, USA). The model was validated by comparing simulation results with field data, revealing the distribution patterns of the velocity field, pressure field, and erosion rate. The study analyzed the impact of nine factors on desander erosion: inlet aspect ratio, cylinder radius, cone length, dust discharge port diameter, exhaust port diameter, particle size, particle concentration, inlet velocity, and operating pressure, clarifying the erosion variation patterns for each factor. SPSSAU V25.0 (Beijing Qingsi Technology Co., Ltd., Beijing, China) was employed to analyze the significance of these nine factors, identifying six significant influencing factors: inlet aspect ratio, cylinder diameter, dust discharge port diameter, particle size, particle concentration, and inlet velocity. Subsequently, response surface analysis was performed using Design-Expert 13 (Stat-Ease, Inc., Minneapolis, MN, USA) to obtain the relationship between the factors and their impact on maximum erosion, leading to the establishment of a predictive model for the maximum erosion rate. In addition, geometry optimization, local wall thickening, coating protection, material selection, and bionic rib structures were discussed as erosion-mitigation strategies. The optimized geometry reduced the erosion rate at the inlet and dust discharge outlet by 20.4% and 21.8%, respectively, while the bionic rib structure reduced the maximum erosion rate by 58%.

## 1. Introduction

During shale gas production, high-velocity gas streams often carry solid particles such as fracturing proppants and formation sand. These particles may cause severe erosion in wellhead equipment, gathering pipelines, and surface facilities, leading to wall thinning, leakage, blockage, and potential safety risks [1,2,3]. Cyclone desanders are therefore commonly installed at shale gas wellheads to remove solid particles before the gas enters downstream facilities. However, the desander itself is continuously exposed to particle-laden swirling flow. Repeated particle–wall impacts can result in localized erosion, shorten equipment service life, and reduce operational reliability. Understanding the erosion behavior of cyclone desanders while maintaining their gas–solid separation performance is therefore essential for safe and stable shale gas production.

Previous studies on cyclone separators have mainly focused on separation efficiency, internal flow structures, and geometric optimization. Classical separation theories established the relationship between separation performance and particle size, residence time, vortex structure, and separator geometry [4,5,6,7]. Later experimental and numerical studies further showed that inlet configuration, cylinder diameter, outlet structure, and operating parameters significantly affect tangential velocity distribution, pressure drop, and separation efficiency [8,9,10,11,12,13]. These studies provide an important basis for cyclone design; however, most of them emphasize separation performance rather than wall erosion. In particular, the erosion behavior of cyclone desanders under high-pressure shale gas wellhead conditions remains insufficiently investigated.

Research on particle erosion has clarified several important damage mechanisms, including micro-cutting, plastic deformation, fatigue damage, and particle rebound effects [14,15,16,17,18,19,20,21,22]. These mechanisms indicate that erosion is governed not only by particle velocity and impact angle, but also by particle size, particle morphology, wall material properties, and particle–wall collision behavior. Although these studies provide useful theoretical support, many of them were conducted using simplified geometries, idealized particle–wall impact conditions, or material-level erosion tests. Their direct applicability to complex swirling flow in shale gas wellhead desanders therefore remains limited.

As computational fluid dynamics (CFD) has matured, the Eulerian–Lagrangian technique has been broadly utilized to characterize gas–solid flow and map erosion-prone regions in pipelines, elbows, valves, and cyclone separators [23,24,25,26,27,28]. CFD models can provide detailed information on velocity distribution, particle trajectories, wall impact locations, and local erosion rates. However, erosion prediction remains sensitive to turbulence closure, particle–wall rebound assumptions, particle morphology, and material parameters. In addition, many numerical studies focus on general pipelines or simplified cyclone structures, while field-validated erosion analysis of shale gas wellhead desanders is still limited. The coupled effects of structural parameters, particle properties, operating conditions, and material response on localized erosion have not yet been fully clarified.

In summary, previous studies have provided valuable theoretical, experimental, and numerical insights into cyclone separation efficiency and particle-induced erosion. However, most existing works focus either on separation performance or on erosion in simplified pipe geometries, whereas systematic erosion analysis of high-pressure cyclone desanders under shale-gas wellhead conditions remains limited. In particular, the coupling among structural parameters, particle properties, operating conditions, material response, and localized wall thinning has not been sufficiently validated using field monitoring data.

To address these gaps, a cyclone desander used at the M shale gas wellhead was investigated in this study. Field data, including gas composition, particle characteristics, operating parameters, and wall-thickness monitoring results, were used to establish and validate a CFD-based erosion model. The effects of structural parameters, particle properties, and operating conditions on the maximum local erosion rate were then systematically analyzed. Significance analysis and response surface methodology were further applied to identify the dominant factors and construct a predictive model. The results provide a basis for understanding erosion mechanisms and developing structural and material-based protection strategies for shale gas wellhead cyclone desanders.

## 2. Field Experimental Study of Cyclone Desanders

### 2.1. M Gas Field Wellsite Process

Cyclone desanders are employed at the M shale gas wellhead for sand removal. The inlet pressure is 38 MPa, the temperature is maintained around 25 °C, the inlet flow rate is 1.6 × 10^5^ m^3^/d, and the inlet velocity is 15 m/s. The separator consists of an inlet, dust discharge port, exhaust port, conical section and cylindrical section. Structural parameters are listed in Table 1.

**Figure 1:**
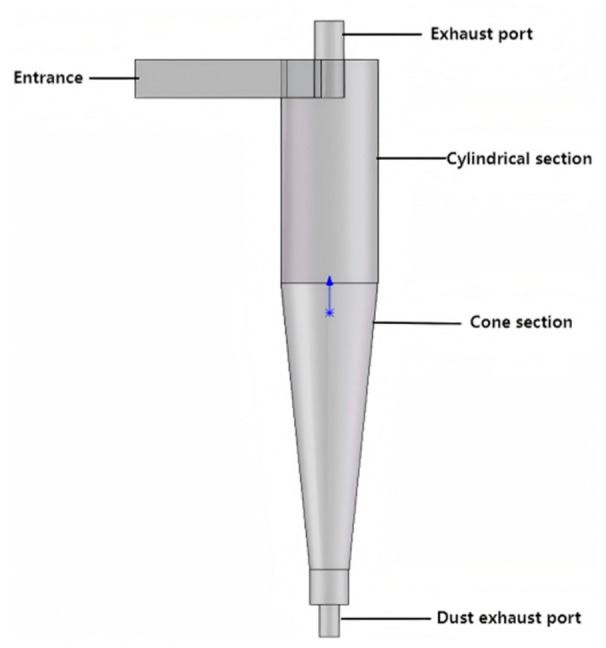
Model Diagram of Cyclone Desander.

As shown in Figure 1, sand particles enter the cyclone separator with the gas stream through the inlet. Inside the separator, most sand particles are driven by inertia and centrifugal force toward the dust outlet for removal, while a small portion is carried out with the purified gas through the exhaust outlet. This process allows the separator to effectively remove sand from the gas stream, thereby protecting downstream equipment.

### 2.2. Gas–Solid Medium Properties

① Gas Properties

Samples were collected from the wellhead using sampling cylinders. The wellhead samples were subjected to gas-chromatography/mass spectrometry (GC-MS) analysis, yielding the components shown in Table 2. C1 and C2 in Table 2 represent methane and ethane, respectively. The gas phase used in the CFD simulation was defined according to the field operating conditions of the M shale gas wellhead.

② Solid Properties

**Figure 2:**
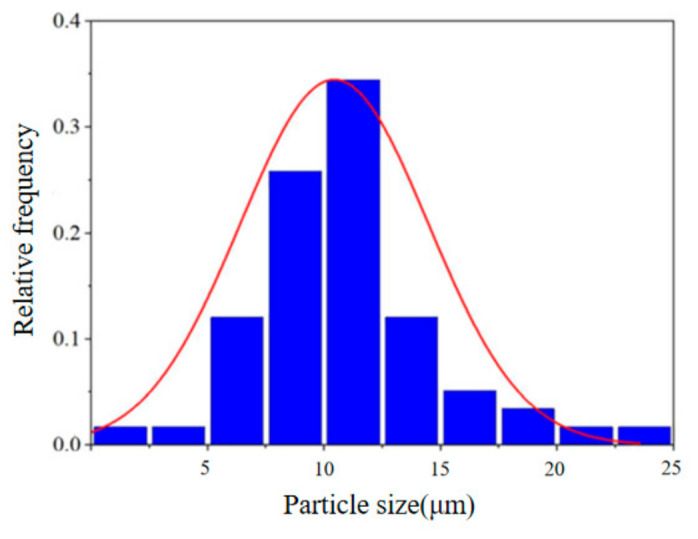
Particle size distribution.

The sand produced from the wellhead of Well M in the shale gas reservoir is predominantly quartz sand, with a field sand production rate of approximately 8 × 10^−4^. Solid particle samples were collected from sediments deposited in the wellhead pipeline. Based on the particle size distribution curve obtained via electron microscopy and statistical analysis of microscopic images, microscopic morphology observation and particle size statistics demonstrate that the average particle size of sand grains is 10 μm, the particle size distribution is shown in Figure 2.

### 2.3. Wall Thickness Monitoring Experiment

**Figure 3:**
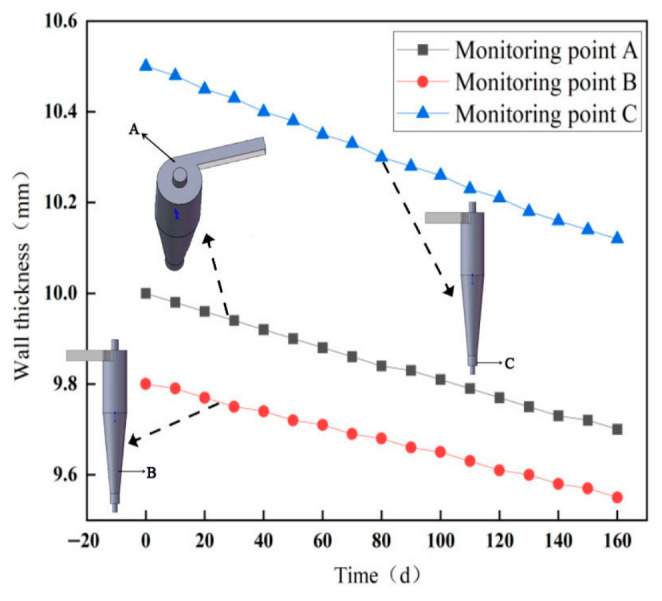
Wall thickness monitoring points and variations.

The WAND wireless passive thickness measurement system was used to monitor wall thickness over a 6-month period, in order to track long-term thinning trends, particularly in scenarios involving gradual corrosion or erosion. Monitoring points A, B, and C were selected at locations prone to severe wear: the inlet, the tapered section, and the dust discharge port. The wall thickness monitoring points and field monitoring data of the sand separator are presented in Figure 3.

The average erosion rates at monitoring points A, B and C were calculated based on the ratio of wall thickness variation to monitoring duration, with the results as follows: 0.00189 mm/d at point A, 0.00156 mm/d at point B, and 0.00239 mm/d at point C. These data provide a fundamental basis for subsequent numerical simulation and validation.

## 3. CFD-Based Numerical Erosion Model for the Cyclone Desander

The present work established a CFD-based numerical erosion model rather than an analytical theoretical model. The gas phase was treated as a steady turbulent continuous phase, and particles were tracked in a Lagrangian framework using the discrete phase model. The particle volume fraction was lower than 10%; therefore, the DPM approach was adopted. The main particle forces considered were drag force and gravity. Particle–wall interaction was described using a reflect boundary condition with normal and tangential restitution coefficients.

### 3.1. Governing Equations

① Continuity Equation

The gas phase was treated as a steady turbulent continuous phase. Therefore, the steady-state continuity equation is given by:

(1) $$\nabla \cdot \left(\rho u\right)=0$$

In the equation, *ρ* is density, kg/m^3^; *u* is the velocity vector, m/s.

② Momentum Equation

The steady-state momentum equation is written as:

(2) $$\nabla \cdot \left(\rho u\times u\right)=-\nabla p+\nabla \cdot \tau +\rho g+S_{m}$$

In the equation, *ρ* is the gas density, u is the gas velocity vector, *p* is the static pressure, *g* is the gravitational acceleration, and *S_m_* is the momentum source term associated with gas–particle interaction. Because a dilute particle loading was considered in the present work, one-way coupling was adopted and Sm was neglected.

The stress tensor τ is defined as:

(3) $$\tau =\mu_{eff}\left[\nabla u+\nabla u^{T}\right]-\frac{2}{3}\mu_{eff}\cdot \nabla u\cdot I$$

In the equation, *μ_eff_* is the effective viscosity, including molecular viscosity and turbulent viscosity, and *I* is the unit tensor.

### 3.2. Particle Motion Equation

Particles were tracked in a Lagrangian framework using the discrete phase model. The particle motion equation can be written as:

(4) $$\frac{du_{p}}{d_{t}}=F_{D}\left(u-u_{p}\right)+\frac{g_{x}\left(\rho_{p}-\rho \right)}{\rho_{p}}+F_{x}$$

(5) FD=18μρpdp2⋅CDRep24

(6) Rep=ρdp|up−u|μ

(7) CD=a1+a2Rep+a3Rep2

In the equation, *u* represents fluid velocity, m/s; *u_p_* denotes particle velocity, m/s; *F_D_* is the drag coefficient; *ρ_p_* is particle density, kg/m^3^; *g_x_* is gravitational acceleration in the X direction, m/s^2^; *Fx* is other forces in the X direction, N; *d_p_* is particle diameter, m; *Re_p_* is the relative Reynolds number; and *C_D_* is the drag coefficient, a function of *Re_p_*, where a_1_, a_2_, and a_3_ are functional coefficients determined by the Reynolds number [29].

In this study, drag force and gravity were considered. Other forces, such as pressure-gradient force, virtual mass force, and Brownian force, were neglected because the particles were relatively large and the gas–solid flow was dominated by inertial transport. The gas phase and particle phase were coupled through particle tracking in the calculated continuous-phase flow field. Because the particle loading was low, one-way coupling was adopted; therefore, particles were affected by the gas flow, while the feedback momentum from particles to the gas phase was neglected.

### 3.3. Erosion Model

The erosion model within ANSYS Fluent within the ANSYS Workbench 19.2 environment (ANSYS, Inc., Canonsburg, PA, USA) is composed of the particle incidence angle, relative velocity, and mass flow rate, expressed as follows:

(8) $$R_{erosion}=\sum_{p=1}^{Nperticle}\frac{m_{p}C\left(d_{p}\right)f\left(\alpha \right)v^{b\left(v\right)}}{A_{face}}$$

In the equation, *R_erosion_* represents the erosion rate, kg/(m^2^·s); *m_p_* denotes the mass flow rate, kg/s; *C(d_p_)* is the particle size function; *f(α)* is the function of the wall impact angle *α*; *b(v)* is the relative velocity function; *A_face_* is the unit erosion area, m^2^; *v* is the relative velocity, m/s; *N_particle_* is the particle number.

### 3.4. Turbulence Models

The RNG k-ε turbulence model was used to describe the turbulent swirling flow inside the cyclone desander. The transport equations for turbulent kinetic energy k and dissipation rate ε are:

(9) $$\frac{\partial \left(\mathrm{pk}\right)}{\partial t}+\frac{\partial \left(pk\mu_{i}\right)}{\partial x_{i}}=\frac{\partial }{\partial xj}\left[\left(\mu +\frac{\mu t}{\sigma k}\right)\frac{\partial k}{\partial xj}\right]+G_{k}-\rho \varepsilon$$

(10) $$\frac{\partial \left(p\varepsilon \right)}{\partial t}+\frac{\partial \left(p\varepsilon \mu_{i}\right)}{\partial x_{i}}=\frac{\partial }{\partial xj}\left[\left(\mu +\frac{\mu t}{\sigma k}\right)\frac{\partial \varepsilon }{\partial xj}\right]+C_{1\varepsilon }\frac{\varepsilon }{k}G_{k}-C_{2\varepsilon }\rho \frac{\varepsilon^{2}}{k}-R_{\varepsilon }$$

In the equation, *G_k_* is the generation of turbulent kinetic energy caused by the mean velocity gradients, *αk* and αε are the inverse effective Prandtl numbers for *k* and *ε*, μeff is the effective viscosity, *Rε* is the additional RNG term, and *Sk* and *Sε* are user-defined source terms. In the present simulation, no additional user-defined source terms were imposed.

### 3.5. Boundary Conditions and Solution Strategy

We developed a CFD-based numerical erosion model for cyclone sand removers using ANSYS Fluent within the ANSYS Workbench 19.2 environment (ANSYS, Inc., Canonsburg, PA, USA). The gas phase is modeled as a stable turbulent continuous phase, while solid particles are tracked using a discrete-phase model within the Lagrangian framework.

The gas inlet was set as a velocity inlet, while the exhaust outlet and dust discharge port were set as outflow boundaries. The wall surface was treated as a no-slip boundary for the gas phase. For the discrete particle phase, the inlet was defined as the particle injection surface, while the exhaust outlet and dust discharge port were set as escape boundaries. The particle–wall boundary was set as “reflect”. The normal and tangential restitution coefficients were defined according to the built-in particle–wall rebound correlation in ANSYS Fluent within the ANSYS Workbench 19.2 environment (ANSYS, Inc., Canonsburg, PA, USA). These coefficients determine the post-impact normal and tangential velocity components and therefore influence the rebound angle, residence time, and secondary particle–wall collisions [30].

### 3.6. Cyclone Desander Mesh Generation

**Figure 4:**
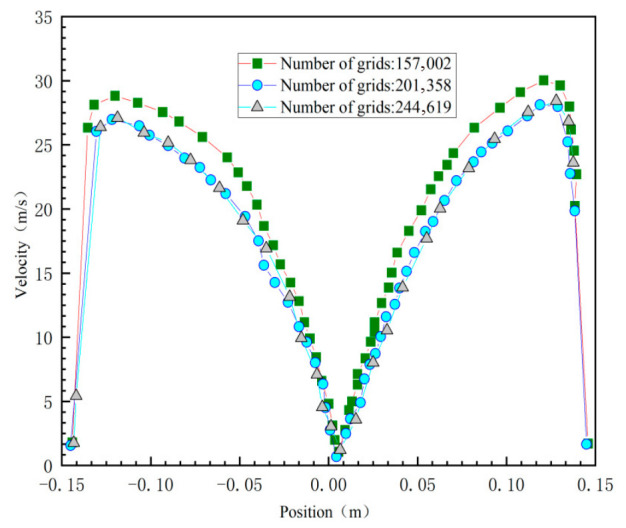
Calculation results of different mesh numbers.

In this study, O-type partitioning was employed for the cyclone desander’s mesh generation. To minimize the impact of mesh density on simulation results, the model mesh was divided into three configurations: 15,700, 20,100, and 24,000 elements. Tangential velocity was used as the metric. Comparative calculations of the tangential velocity at the x = 0 cross-section were performed to determine the optimal mesh count for subsequent simulations. Figure 4 illustrates the influence of different mesh counts on the tangential velocity distribution at the x = 0 cross-section of the cyclone desander. The figure indicates that simulation results exhibit good consistency when the mesh sizes are 201,358 and 244,619. However, when the mesh size is 157,002, the discrepancy with the other two mesh sizes becomes significant. Therefore, to balance computational efficiency and result accuracy while ensuring the reliability of the research findings, this study ultimately selected a mesh size of 201,358 for subsequent numerical simulation analysis.

For particle–wall interaction, the wall boundary was set as “reflect”. The normal and tangential restitution coefficients were used to calculate the post-impact velocity of particles after collision with the wall. These coefficients affect the rebound angle, residence time, particle trajectory, and local erosion rate. In the baseline model, the wall material was treated as carbon steel, and the corresponding restitution-coefficient correlations were adopted.

### 3.7. Verification of Sand Separator Model

**Figure 5:**
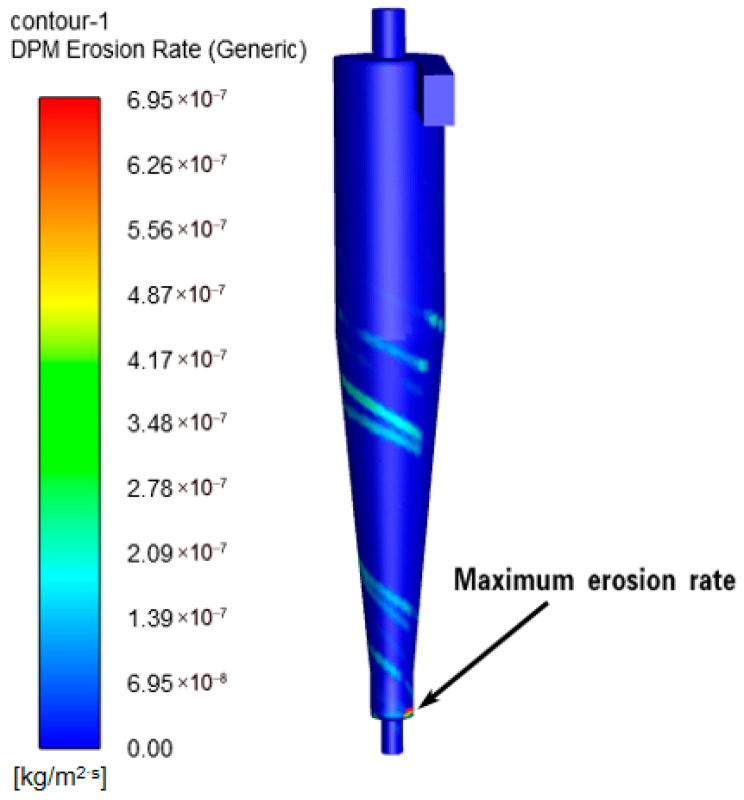
Erosion cloud of cyclone desander.

Simulations using ANSYS Fluent within the ANSYS Workbench 19.2 environment (ANSYS, Inc., Canonsburg, PA, USA) revealed the erosion patterns of the cyclone desander, as shown in Figure 5. The erosion zones were primarily identified at the inlet, conical section, and bottom outlet. The erosion model outputs the mass loss rate per unit wall area, with a unit of kg/(m^2^·s). For comparison with field wall-thickness reduction, it was converted into an equivalent thinning rate using the material density. However, the unit of wall thickness reduction for the wellhead desander is mm/y. The conversion formula between these units is shown in Equation (11).

(11) $$M_{erosion}=\frac{A_{erosion}\times 3600\times 24\times 360}{\rho_{C}}$$

In the equation, $M_{\mathrm{erosion}}$ denotes the wellbore wall thinning rate, mm/y; $A_{\mathrm{erosion}}$ represents the erosion rate, kg/m^2^·s. $\rho_{C}$ is the material density, kg/m^3^, taken as 7850 kg/cm^3^.

**Figure 6:**
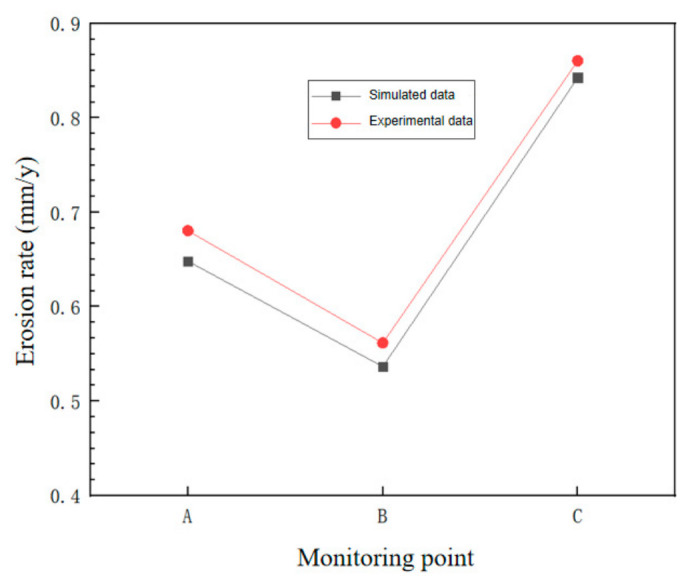
Comparison of simulated data and field data.

To ensure the accuracy of the simulated erosion rate, the simulated erosion rate was compared with the wall thickness reduction rate obtained from field measurements. The results are compared as shown in Figure 6.

The relative simulation errors at monitoring points A, B, and C were 4.73%, 4.45%, and 2.06%, respectively, with an average relative error of 3.75%. This indicates that the CFD-based erosion model can reasonably predict the main erosion-prone regions of the cyclone desander. To improve the reliability of the validation, uncertainty sources associated with field wall-thickness monitoring and numerical prediction were further considered. The WAND wireless passive wall-thickness monitoring system has a measurement resolution of 0.01 mm. The experimental uncertainty mainly originated from measurement resolution, installation repeatability, local wall roughness, and fluctuations in sand production rate. The numerical uncertainty was mainly related to mesh resolution, boundary-condition simplification, particle-size distribution, turbulence closure, and empirical constants in the erosion model. Therefore, although the validation results are supported by field monitoring data, they should still be interpreted as case-dependent.

### 3.8. Model Assumptions and Limitations

Although the CFD-based erosion model was validated using field wall-thickness monitoring data, several assumptions and limitations should be noted. First, the gas phase was treated as a steady turbulent continuous phase. Therefore, transient fluctuations of the vortex core, unsteady particle clustering, and short-term sand-production fluctuations were not fully resolved. Second, the discrete phase model tracks particles in a Lagrangian framework and is suitable for dilute particle-laden flow; however, particle–particle collisions and particle agglomeration were not explicitly considered. This may influence the predicted particle trajectories and local impact frequency, especially under high particle-concentration conditions.

Third, particle–wall collision was described using a reflect boundary condition with normal and tangential restitution coefficients. In practical operation, the rebound behavior may be affected by particle shape, particle hardness, wall roughness, local plastic deformation, and erosion-induced surface damage. These factors were simplified in the present model. Fourth, the erosion model evaluates wall material loss mainly from impact velocity, impact angle, particle mass flow rate, and particle size. The possible coupling between erosion and corrosion, as well as changes in material properties caused by long-term service, was not included.

In addition, uncertainty remains due to wall-thickness measurement accuracy, local wall roughness, particle-size distribution, mesh resolution, turbulence closure, and empirical constants in the erosion model. Therefore, the present model is more appropriate for identifying erosion-prone regions and comparing relative trends under different structural and operating parameters than for providing an absolute lifetime prediction for all field conditions. Future work should include more monitoring points, longer-term wall-thickness measurements, material-level erosion tests, and erosion–corrosion coupling analysis to further improve the predictive capability of the model.

## 4. Analysis of Factors Affecting Erosion in Cyclone Desanders

### 4.1. Influence of Structural Parameters

All single-factor simulations in Section 4.1, Section 4.2 and Section 4.3 were conducted based on the original cyclone desander geometry, with only one parameter varied at a time. Therefore, the values obtained from the single-factor analysis represent preferred candidate values under isolated parameter variation, rather than the final optimized structure. The final optimized dimensions were determined later using response surface methodology under coupled multi-factor conditions.

#### 4.1.1. Effect of Inlet Aspect Ratio on Wall Erosion in Cyclone Desanders

To investigate the effect of inlet aspect ratio on wall erosion in cyclone desanders, this study conducted comparative analyses by adjusting only the inlet aspect ratio while keeping all other structural parameters constant. Referencing existing research, the inlet area was fixed at the value corresponding to an aspect ratio of approximately 1.5 to ensure comparability of results. Specific dimensions are shown in Table 3.

**Figure 7:**
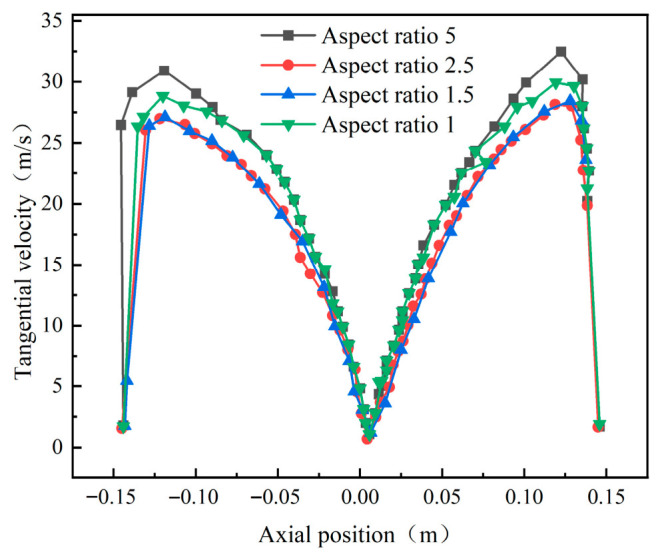
Tangential velocity curves at different inlet aspect ratios at y = 200.

Before analyzing wall erosion characteristics, the internal flow field within the cyclone desander was first investigated under different inlet aspect ratios. Figure 7 shows the tangential velocity distribution at the y = 200 mm cross-section of the conical component. Results reveal a symmetrical internal tangential velocity distribution: low-velocity zones exist in both the outer and central regions, separated by a high-velocity band. When the aspect ratio is 5, the tangential velocity peaks at 33 m/s; as the aspect ratio decreases, the tangential velocity correspondingly diminishes.

**Figure 8:**
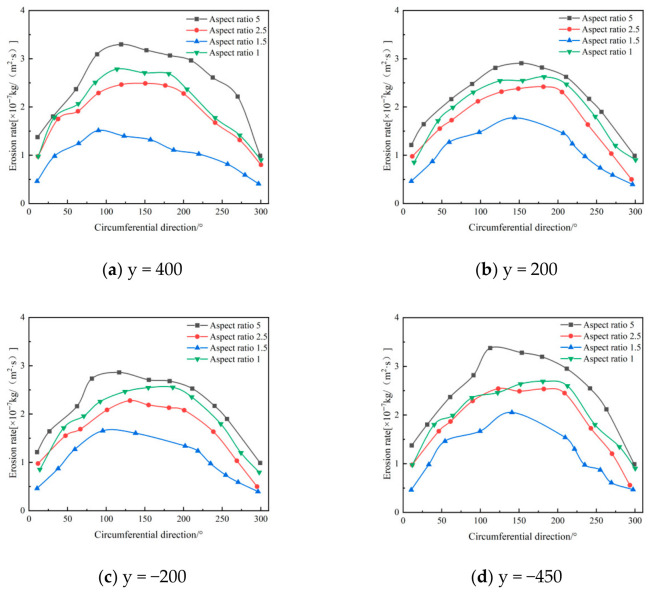
Effect of different height-width ratios on wall erosion in annular region.

Figure 8 depicts the circumferential distribution of erosion rates along the inner wall surface within the annular region. At different axial positions, the erosion rate distribution trends are similar across various aspect ratios, exhibiting a pattern of initial increase followed by decrease, consistent with numerical simulation results. As the axial position increases, the maximum erosion rate gradually decreases, with erosion concentrated in the 90° to 270° range. When the aspect ratio increases from 1.5 to 2.5, the erosion rate within the annular region changes little, especially at the 400 mm and −450 mm positions, where the curves for aspect ratios of 2.5 and 1.5 closely match.

**Figure 9:**
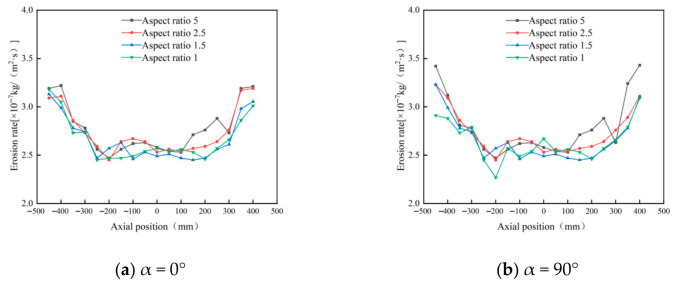

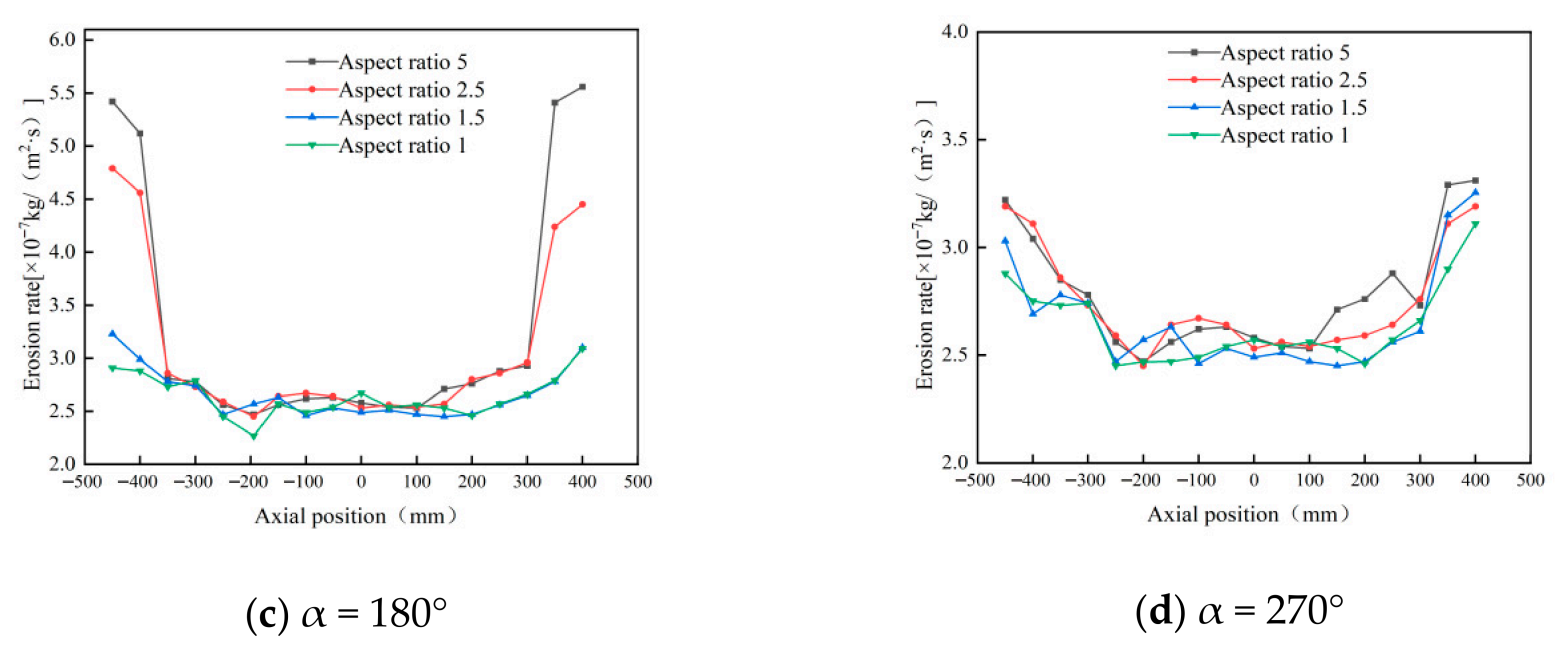
Effect of different inlet aspect ratios on the erosion of cyclone walls.

Figure 9 illustrates the axial variation in erosion rates at different wall angle configurations. Under all operating conditions, erosion rates exhibit a pattern of first decreasing and then increasing from top to bottom. When the inlet wall’s annular angle is 180°, significant differences in erosion rates are observed across varying aspect ratios: at an aspect ratio of 5, the top erosion rate reaches 5.45 × 10^−7^ kg/(m^2^·s), whereas at an aspect ratio of 1, it drops to only 2.96 × 10^−7^ kg/(m^2^·s). This indicates that a larger aspect ratio leads to more severe erosion. The primary reason is that an increased aspect ratio enhances the tangential velocity, thereby increasing the rotational kinetic energy of particles and intensifying erosion in both the inlet and dust discharge regions [31].

**Figure 10:**
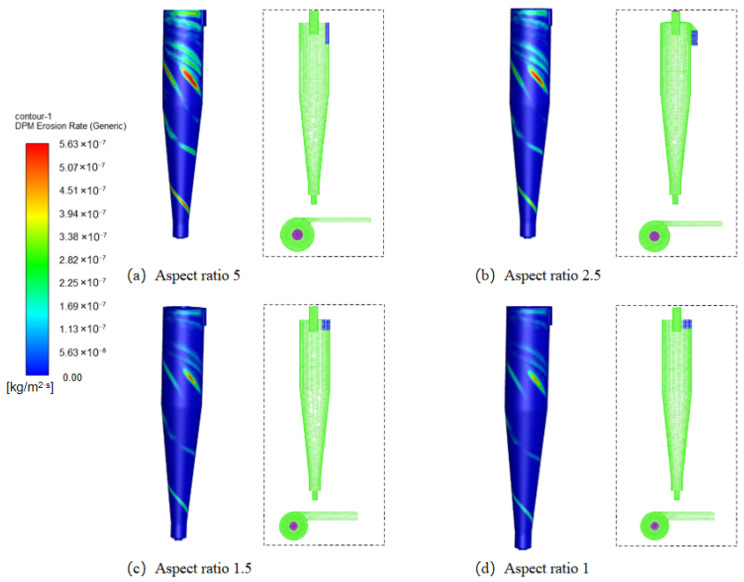
Erosion of cylinders with different aspect ratios.

Figure 10 further compares erosion patterns on the cylinder body under different aspect ratios. The most pronounced erosion occurs at an aspect ratio of 5, with a maximum erosion rate of 5.45 × 10^−7^ kg/(m^2^·s), followed by an aspect ratio of 2.5. Erosion levels at aspect ratios of 1.5 and 1 were comparable and relatively mild. Research indicates that cyclone desander inlet aspect ratios between 2 and 4 can balance the smooth entry of gas flow with internal vortex formation, thereby enhancing sand removal efficiency [32].

In summary, an appropriate aspect ratio helps reduce erosion while maintaining separation efficiency. The aspect ratio of 2.5 used in this study meets practical engineering requirements, effectively mitigating erosion with minimal impact on separation performance. In actual applications, the aspect ratio can be adjusted based on equipment model, gas characteristics, and separation requirements.

#### 4.1.2. Effect of Cylinder Radius on Wall Erosion of Cyclone Desanders

**Figure 11:**
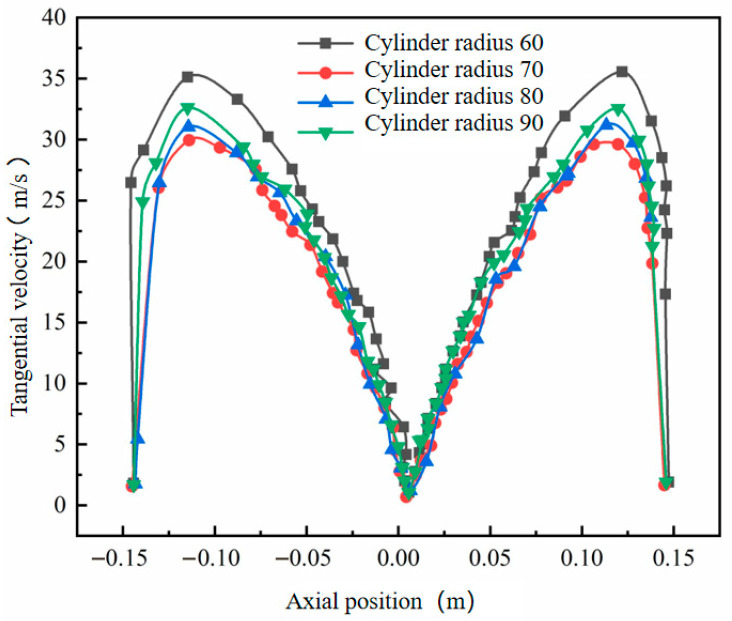
Tangential velocity curves with different cylinder radii at y = 200.

Using the original cylinder radius of 75 mm as the baseline, four additional cylinder radii—60 mm, 70 mm, 80 mm, and 90 mm—were studied. Prior to analyzing wall erosion characteristics, numerical simulations were conducted to examine internal flow field properties under different cylinder diameters. Analysis of the data in Figure 11 reveals that while the cylinder radius varies, its impact on the tangential velocity of the outer vortex is limited. However, in the conical region (y = 200 mm), the maximum tangential velocity gradually decreases as the cylinder radius increases. This change directly results in a significant reduction in the kinetic energy carried by particles passing through the conical region.

**Figure 12:**
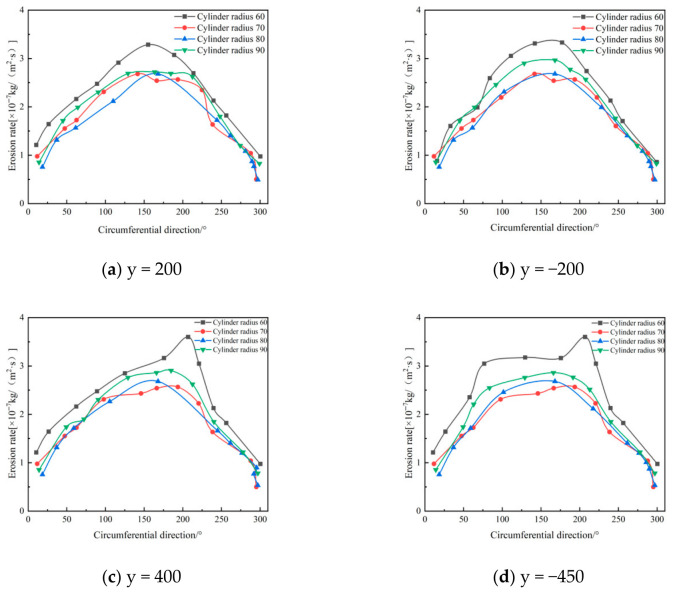
Effect of different cylinder diameters on the erosion of annular region walls.

**Figure 13:**
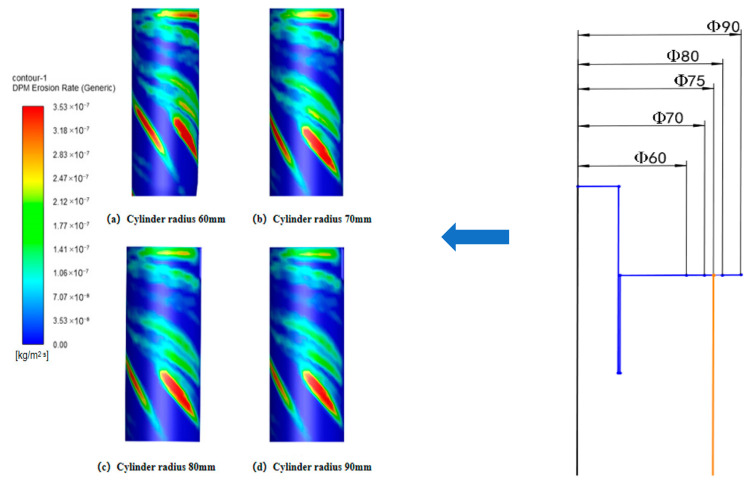
Erosion contours of cylinder wall under different cylinder radii.

**Figure 14:**
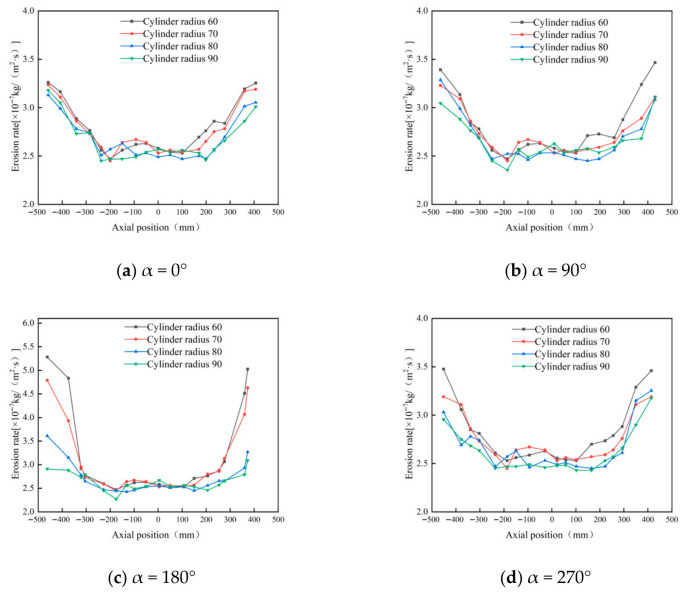
Effect of different cylinder radii on wall erosion of cyclone separator.

Figure 12, Figure 13 and Figure 14 show that changing the cylinder radius mainly affects the erosion intensity rather than the overall circumferential distribution. The high-erosion region remains concentrated near the inlet and dust outlet, especially within the 90–270° sector, indicating that inlet-induced swirling flow controls the main particle–wall impingement pattern. When the cylinder radius is small, the gas–solid flow is confined within a narrower chamber, which increases tangential velocity and radial velocity gradients. The stronger vortex fluctuation increases particle residence near the wall and raises both impact frequency and impact energy, resulting in more severe local erosion, particularly at a radius of 60 mm. Increasing the radius weakens the near-wall swirl and reduces the peak erosion rate; however, the difference between 80 mm and 90 mm is limited, with maximum erosion rates of 3.21 × 10^−7^ and 3.15 × 10^−7^ kg/(m^2^·s), respectively. Therefore, considering erosion mitigation, compact structure, and separation performance, a cylinder radius of 80 mm was selected as the preferred value [33].

#### 4.1.3. Effect of Cone Length on Wall Erosion of Cyclone Desanders

Since the cylinder radius and cone radius vary synchronously, the influence of the cone radius on the erosion rate is not investigated separately. This study therefore focuses on the effect of cone length on wall erosion. Using the original cone length of 350 mm as the baseline, three additional lengths—330 mm, 310 mm, and 290 mm—were considered.

**Figure 15:**
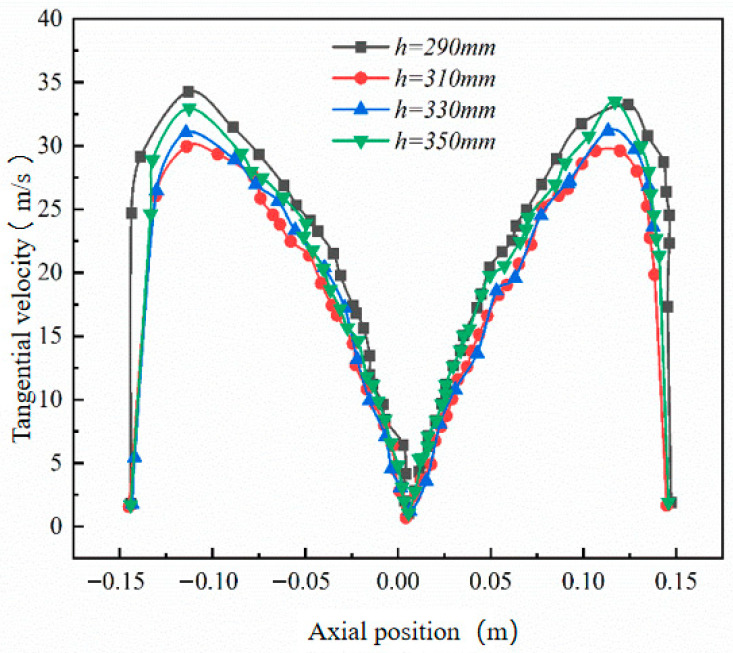
Tangential velocity curves for different cone lengths at y = 200.

Figure 15 shows that increasing the cone length reduces the peak tangential velocity in the conical section. A longer cone provides a larger deceleration and separation space, allowing the gas–solid flow to develop a smoother downward spiral. As a result, particles strike the cone wall with lower kinetic energy, which helps reduce local erosion.

**Figure 16:**
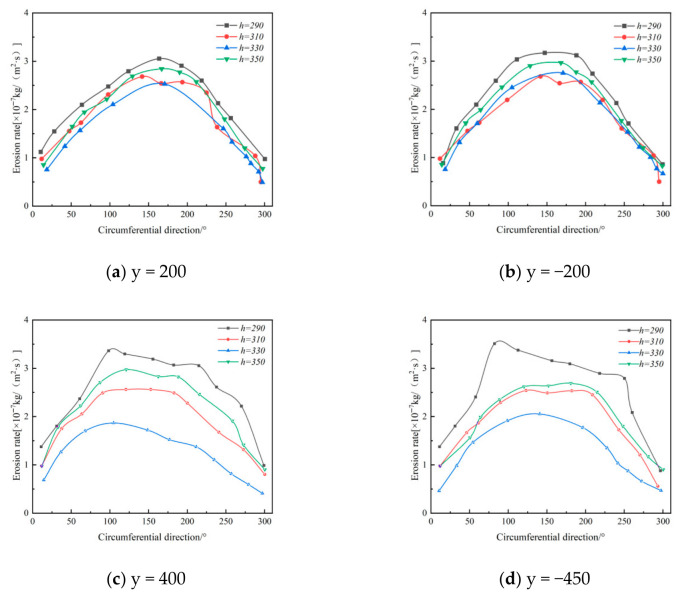
Effect of different cone lengths on wall erosion in annular region.

**Figure 17:**
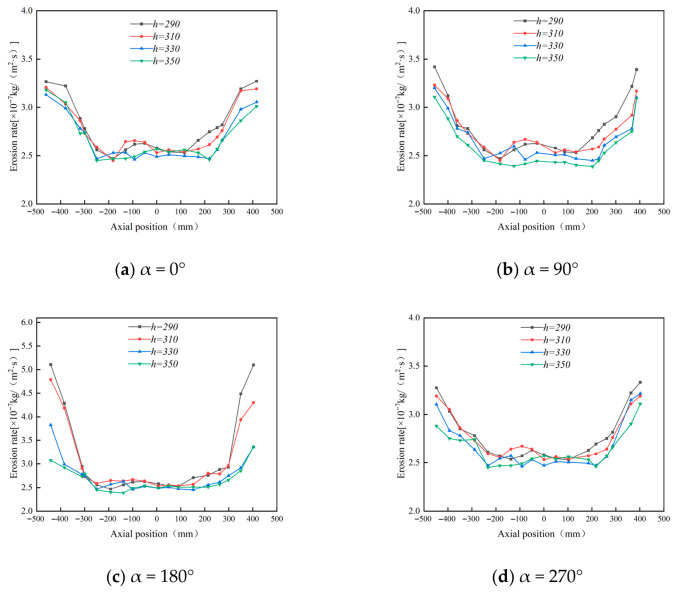
Effect of different cone lengths on wall erosion of cyclone separator.

Figure 16 and Figure 17 indicate that cone length changes the magnitude of erosion more significantly than its circumferential distribution. The main erosion zones remain near the inlet and dust outlet, especially within the 90–270° sector. A shorter cone compresses the swirling flow, strengthens near-wall acceleration, and increases particle impingement at the lower cone and dust outlet. As the cone length increases, the particle impact velocity and impact frequency decrease. For example, at the 180° annular angle, the maximum erosion rate decreases from 5.24 × 10^−7^ to 3.14 × 10^−7^ kg/(m^2^·s), confirming that a longer cone can effectively suppress local erosion.

**Figure 18:**
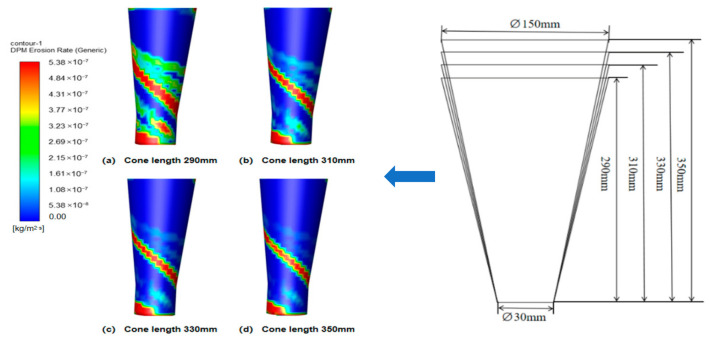
Erosion of the bottom end of the cone by different cone lengths.

Figure 18 further confirms that the 290 mm cone produces the most severe lower-cone erosion, whereas the 330 mm and 350 mm cases show similar and lower erosion levels. Considering both erosion mitigation and separation stability, 350 mm was selected as the preferred cone length.

#### 4.1.4. Effect of Dust Outlet Diameter on Wall Erosion in Cyclone Desanders

**Figure 19:**
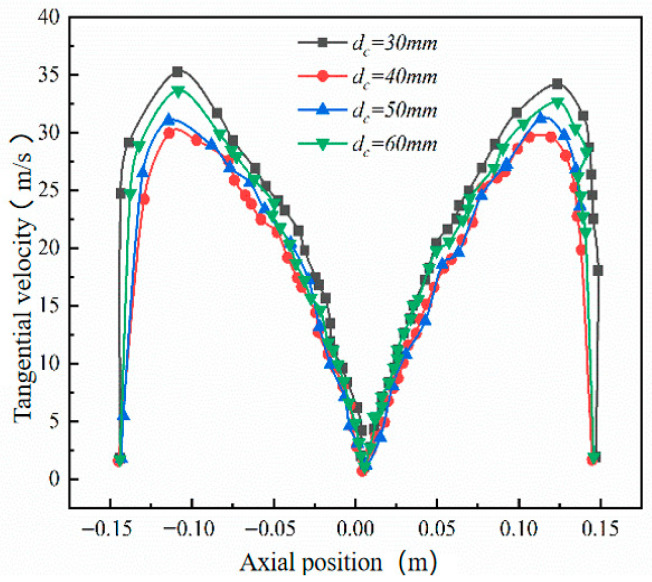
Tangential velocity curves of different dust discharge port diameters at y = 200.

Building on experiments with the original separator structure, this study investigated dust outlet diameters of 40 mm, 50 mm, and 60 mm to clarify their effect on wall erosion patterns, and conducted corresponding numerical simulations of the internal flow field. Figure 19 shows the tangential velocity distribution at the y = 200 mm cross-section of the cone for different dust outlet diameters. As the outlet diameter increases, the maximum tangential velocity at this cross-section decreases.

**Figure 20:**
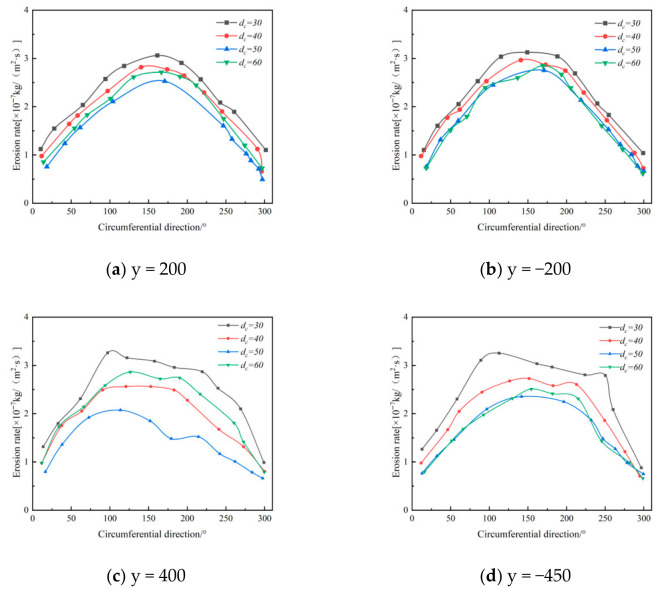
Effect of Different Dust Discharge Port Diameters on Wall Erosion of the Annular Space.

Figure 20 shows the circumferential distribution of wall erosion rates at various axial positions within the annular region of the desander. The results indicate that changes in the dust discharge port diameter have little effect on this distribution pattern, which remains consistent across all axial positions. Furthermore, the maximum local erosion rate gradually decreases with increasing axial height, and the peak erosion at each position is consistently concentrated within the 90–180° range.

**Figure 21:**
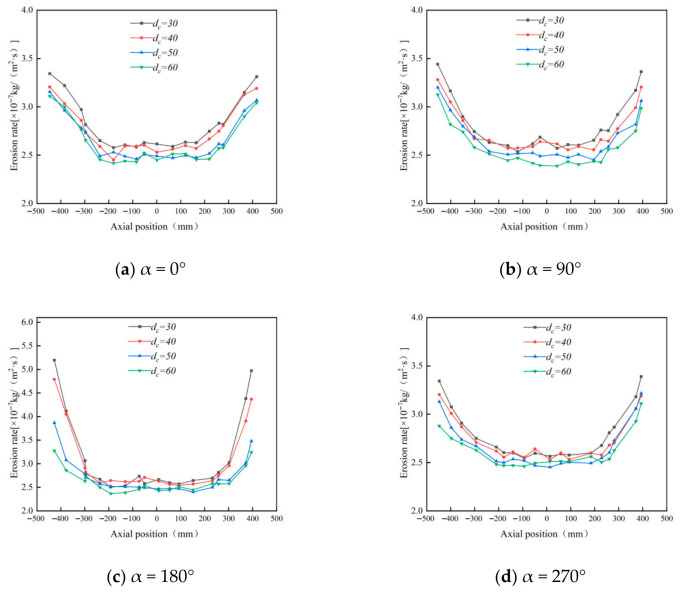
Effect of Different Dust Discharge Port Diameters on Wall Erosion.

Figure 21 shows how erosion rates vary with axial position at different annular angles. The maximum erosion consistently occurs near the inlet and the dust discharge port. At annular angles of 90° and 180°, the effect of the dust discharge port diameter on erosion is particularly evident in these regions. Furthermore, as the outlet diameter increases, erosion severity in the most affected areas decreases significantly, with the maximum erosion rate falling from 5.34 × 10^−7^ kg/(m^2^·s) to 3.40 × 10^−7^ kg/(m^2^·s).

Figure 21 further shows that erosion is most severe with a dust outlet diameter of 30 mm, reaching a maximum rate of 5.39 × 10^−7^ kg/(m^2^·s). At 60 mm, the maximum erosion rate drops to 3.45 × 10^−7^ kg/(m^2^·s), while erosion levels for diameters of 40 mm and 50 mm are similar. If sand removal efficiency is the primary goal, a 40 mm outlet is more suitable for this separator model; if minimizing erosion is prioritized, the 50 mm outlet is preferable. The final optimal diameter will be determined through subsequent multi-factor analysis.

#### 4.1.5. Effect of Exhaust Port Diameter on Wall Erosion of Cyclone Desanders

The exhaust pipe is a core component of cyclone desanders, serving as the outlet for purified gas after gas–solid separation [34]. Based on the original structure, this study examines three exhaust port diameters (*dr*): 50 mm, 55 mm, and 60 mm, to investigate their effect on erosion characteristics.

**Figure 22:**
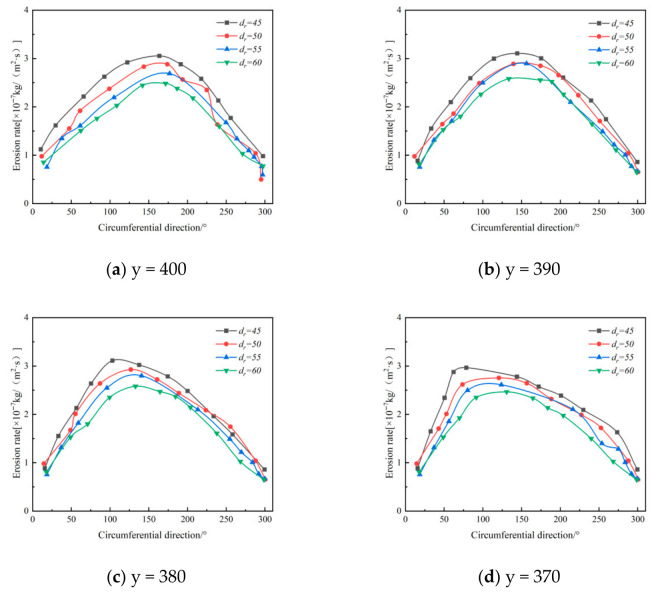
Effect of different exhaust port diameters on wall erosion in annular region.

Figure 22 shows the circumferential distribution of wall erosion rates for different values of *dr*. The results indicate that regardless of *dr*, the erosion rate at each axial position in the annular region follows a circumferential pattern of initially increasing and then decreasing, consistent with local erosion behavior in this zone. Areas of severe erosion are concentrated between 0° and 180°, with the most intense erosion occurring between 60° and 180°.

Comparing different axial positions reveals that erosion rates gradually increase as the axial position shifts downward (i.e., as the position value decreases). When the *dr* value decreases (meaning the radial size of the exhaust port is reduced), the erosion rate in the upper 0–60° section of the annular region remains largely stable, whereas the erosion rate in the 50–300° section rises significantly. For example, when *dr* is reduced from 60 mm to 45 mm, the erosion peak at the 370 mm axial position shifts from 100° to 90°. While the peak erosion location in the upper part of the exhaust port stays stable, the area of severe erosion expands; conversely, the peak location in the lower part shifts noticeably with changes in *dr*. In summary, the value of *dr* significantly affects the distribution, peak location, and extent of erosion on the annular wall, producing distinct erosion patterns at different axial positions. Adjusting the exhaust port diameter substantially alters the flow behavior in the annular region: although the basic profile of tangential velocity remains similar, a smaller *dr* markedly increases the maximum tangential velocity. This increase raises the kinetic energy of solid particles striking the wall, directly leading to higher local erosion rates [35]. Reducing the exhaust port size also weakens the confinement effect exerted by the outer wall of the exhaust pipe on the particles, altering their trajectories. As a result, not only does the contact point between particles and the wall move rearward, but the transition from linear to curved motion is displaced, ultimately increasing the azimuthal angle corresponding to the maximum erosion rate. During gas–solid separation, a highly concentrated particle band forms near the lower wall of the desander, with particles descending along high-speed spiral paths. This motion pattern further shapes the erosion distribution. Specifically, a smaller *dr* intensifies the fluid centrifugal force. Higher tangential velocity enhances the centrifugal force acting on particles, driving them toward the wall and increasing the frequency of particle–wall interactions, which consequently intensifies erosion and wall wear [36].

**Figure 23:**
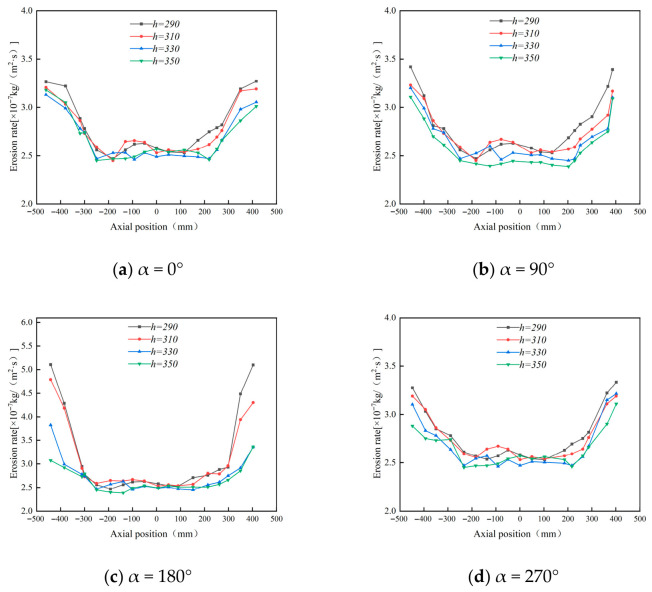
Effect of Different Exhaust Port Diameters on Wall Erosion.

Figure 23 shows that among the erosion rates at different axial positions, the 90° and 180° positions exhibit the most significant influence from the exhaust port diameter, with the maximum erosion rate decreasing from 5.21 × 10^−7^ kg/(m^2^·s) to 3.41 × 10^−7^ kg/(m^2^·s).

Under otherwise constant operating conditions, a smaller exhaust port diameter is associated with higher separation efficiency in the desander. Considering both erosion characteristics and separation efficiency, a diameter of 50 mm is determined to be optimal.

### 4.2. Influence of Medium Parameters

#### 4.2.1. Effect of Particle Size on Wall Erosion in Cyclone Desanders

**Figure 24:**
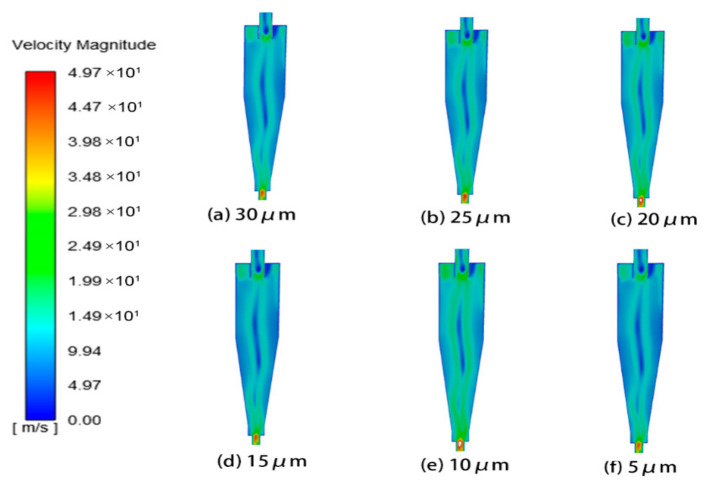
Cyclone velocity field with different particle sizes.

**Figure 25:**
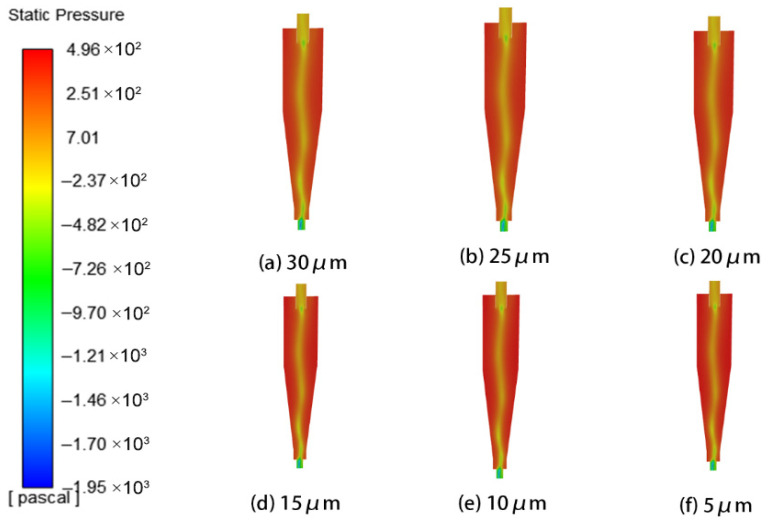
Cyclone pressure field with different particle sizes.

This study systematically investigated the influence of particle size on erosion characteristics using six sizes: 5 μm, 10 μm, 15 μm, 20 μm, 25 μm, and 30 μm. Analysis of the pressure and velocity fields within the cyclone separator revealed that as particle size increased, the velocity at the inlet and outlet followed a trend of first increasing and then decreasing, whereas the pressure field showed no significant change. The corresponding flow field distributions are presented in Figure 24 and Figure 25.

**Figure 26:**
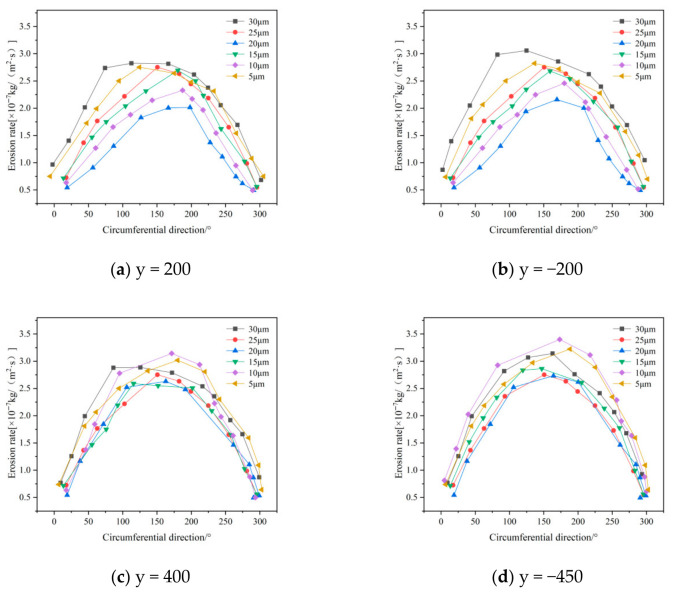
Effect of different particle sizes on wall erosion in annular region.

The wall erosion rates at various axial positions in the annular region all follow a circumferential distribution pattern that initially increases and then decreases. However, the peak location varies with particle size. For particle sizes of 5, 10, and 15 μm, the erosion peaks are concentrated near 180°, whereas at 30 μm, the peak shifts to approximately 90° (Figure 26). In the axial regions of the cylinder and cone, erosion rates generally increase with particle size. This trend occurs because larger particles possess greater mass, which leads to stronger centrifugal forces during rotation. As a result, particle–wall collisions become more energetic, intensifying erosion [37]. It should be noted, however, that at the inlet and outlet positions of the desander, the peak erosion rate for 10 μm particles is higher than that for larger sizes such as 20–30 μm.

**Figure 27:**
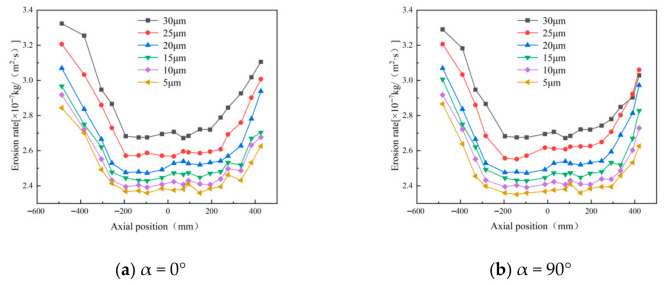

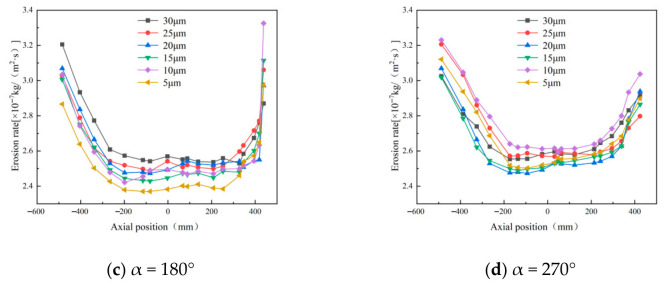
Effect of different particle sizes on wall erosion of cyclone separator.

The erosion distribution on the desander wall surface for different particle sizes (Figure 27) indicates that the overall erosion rate increases with particle size. The location of the maximum erosion rate remains consistent across all particle sizes, suggesting that the wear on the cylinder exhibits a spiral distribution pattern. In the conical section, at a given circumferential position, the erosion rate first increases and then decreases as the axial position moves downward toward the dust discharge port, with the peak occurring at the port itself. Notably, the peak erosion rates for smaller particles (5–10 μm) are significantly higher than those for larger particles (25–30 μm), while larger particles show a more uniform erosion distribution at the same axial position.

**Figure 28:**
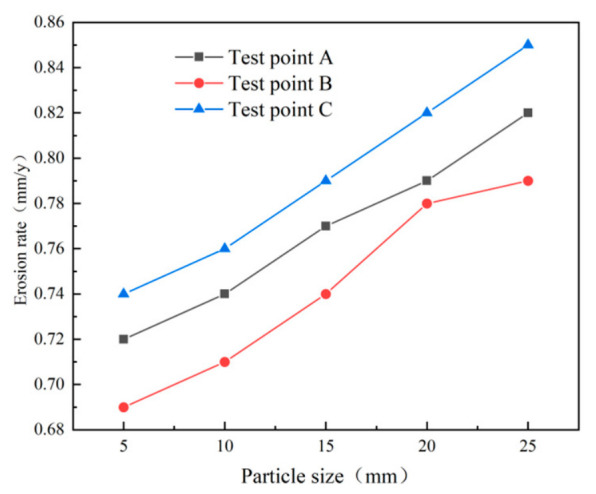
Erosion rate plot of monitoring points.

**Figure 29:**
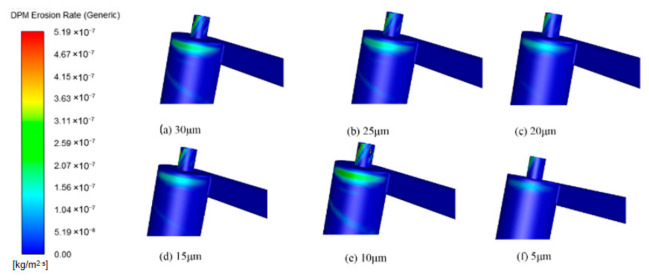
Erosion at the inlet under different particle sizes.

**Figure 30:**
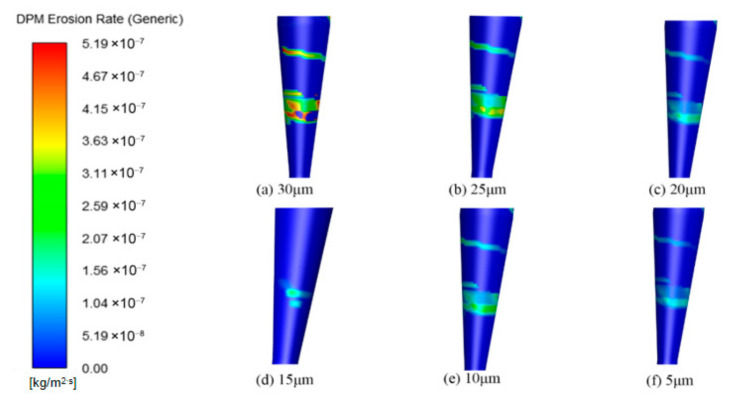
Cone erosion at different particle sizes.

**Figure 31:**
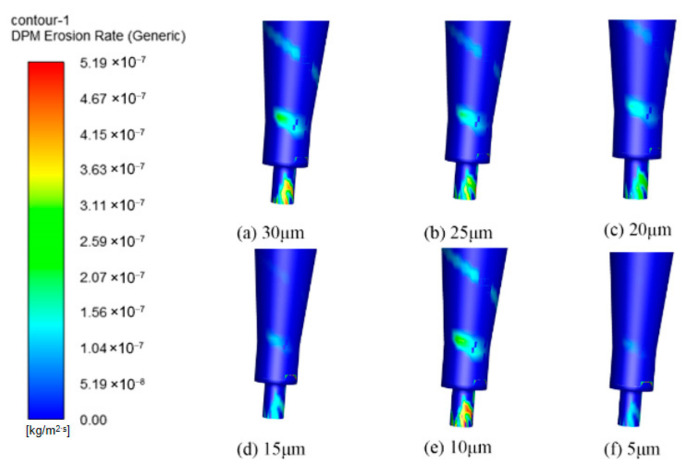
Erosion at dust outlets of different particle sizes.

Erosion simulation results (Figure 28) show that the erosion rates at all three monitoring points increase with particle size, with larger particles producing higher erosion rates. The erosion rate at the monitoring points shows a gradual increase as the particle diameter rises. As shown in Figure 29, the most serious wall erosion and the largest erosion area at the cyclone desander inlet occur under the particle size of 10 μm. The key erosion characteristics differ across sections (Figure 30 and Figure 31). At the inlet, 10 μm particles caused the most severe wall erosion and the largest eroded area. In the conical section, 30 μm particles produced the most intense localized erosion, and the erosion rate in this region increased with particle size. At the dust outlet, erosion caused by both 10 μm and 30 μm particles was comparatively severe, with the maximum erosion rate for 10 μm particles slightly exceeding that for 30 μm particles.

#### 4.2.2. Effect of Particle Concentration on Wall Erosion in Cyclone Desanders

**Figure 32:**
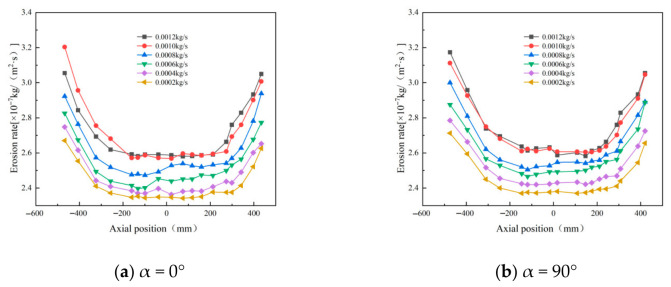

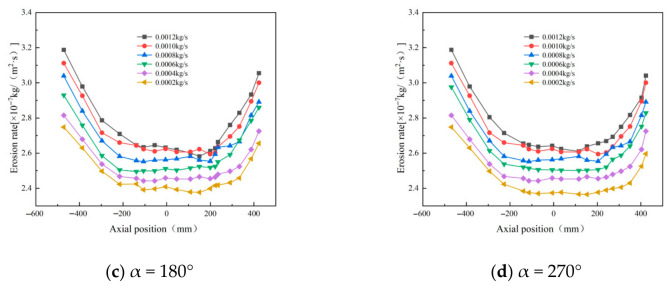
Effect of different particle concentrations on wall erosion in annular region.

Wall erosion in cyclone separators is closely linked to the inlet particle concentration and shows a positive correlation with separation efficiency—higher concentrations typically lead to better separation. To isolate its effect, the sand flow rate at the inlet was varied while all other parameters were held constant. Inlet mass flow rates of 0.0002, 0.0004, 0.0006, 0.0008, 0.001, and 0.0012 kg/s were used to study the resulting erosion characteristics. Figure 32 shows the erosion patterns of the cyclone separator under different concentrations.

As shown in Figure 32, the wall erosion rate increases with particle concentration in all four directions analyzed. At the highest concentration (0.0012 kg/s), the peak erosion occurs at α = 0°. Notably, the maximum erosion value alternates between pronounced peaks and troughs. At lower concentrations, the erosion rate along the conical wall changes gradually and remains relatively stable. As concentration increases, enhanced particle agglomeration during sand removal leads to smaller particles being entrained by larger ones, which improves separation efficiency [38]. However, excessively high concentrations increase inter-particle collisions and promote oscillatory particle motion, ultimately reducing the separation efficiency of the cyclone separator.

**Figure 33:**
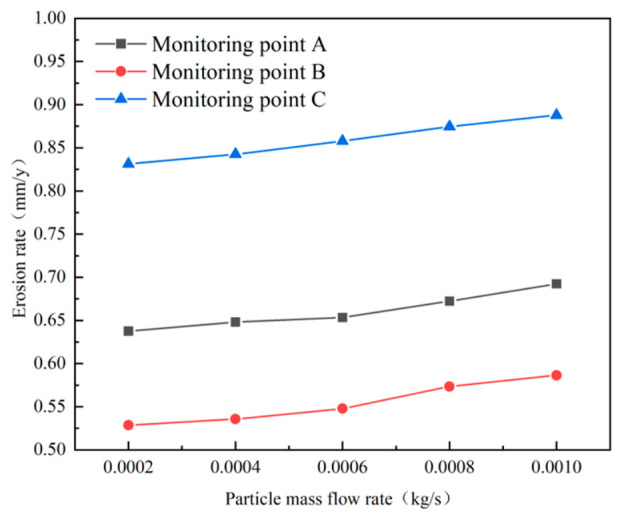
Variation in erosion rate at monitoring points at different mass flow rates.

Simulations performed using ANSYS Fluent within the ANSYS Workbench 19.2 environment (ANSYS, Inc., Canonsburg, PA, USA) show the erosion patterns at monitoring points A, B, and C under different flow conditions (Figure 33). The results indicate that erosion at these points increases with particle concentration, confirming a positive correlation between wall erosion in the cyclone separator and the level of particle concentration.

### 4.3. Influence of Operating Parameters

#### 4.3.1. Effect of Inlet Velocity on Wall Erosion of Cyclone Desanders

**Figure 34:**
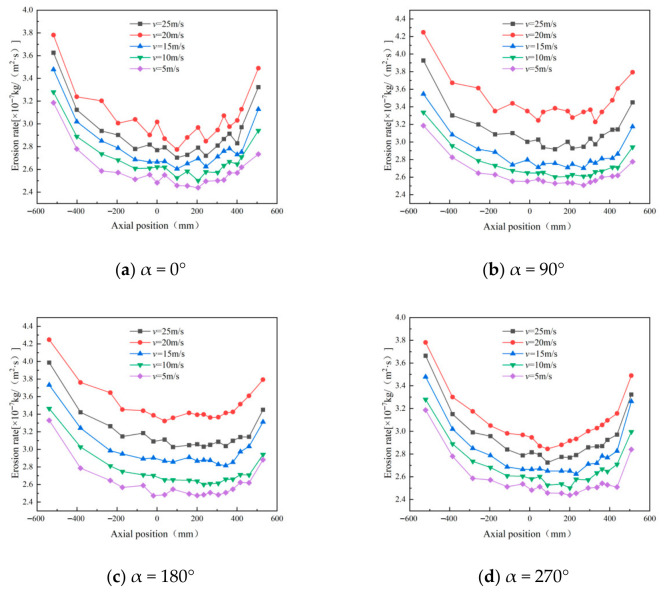
Effect of different inlet velocities on wall erosion of cyclone separator.

The inlet velocity is a key parameter affecting both wall erosion and the operational performance of cyclone separators [39]. In this study, the erosion process was simulated for five inlet velocities: 5 m/s, 10 m/s, 15 m/s, 20 m/s, and 25 m/s. A relationship curve between erosion rate and velocity was plotted (Figure 34). The results indicate that the erosion rate on the separator wall is positively correlated with inlet velocity, with the most severe erosion occurring at the dust discharge port. When the inlet velocity increased from 5 m/s to 25 m/s, the wall erosion rate rose from 3.86 × 10^−7^ kg/(m^2^·s) to 4.28 × 10^−7^ kg/(m^2^·s). The underlying mechanism is that the incident velocity of the solid particles is aligned with the gas inlet velocity. An increase in inlet velocity imparts greater centrifugal force to the particles, thereby intensifying their impact and the resulting erosion on the wall surface.

**Figure 35:**
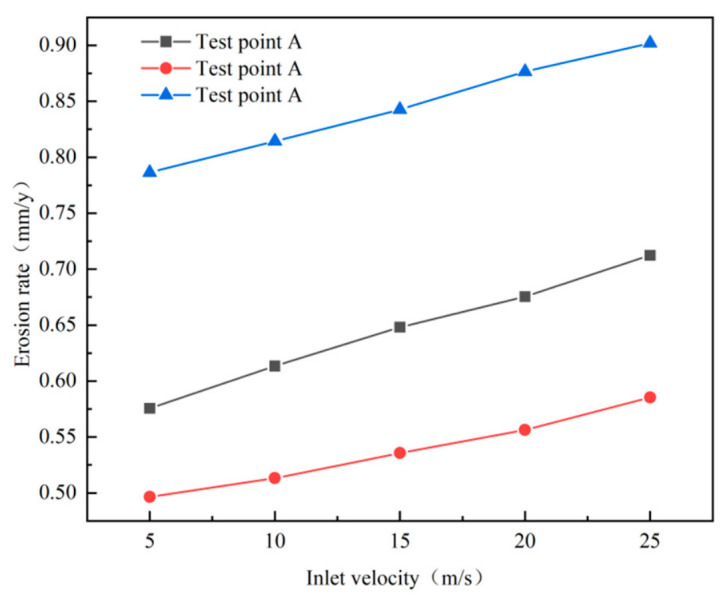
Variation in erosion rate at monitoring points at different inlet velocities.

Simulated erosion data for monitoring points A, B, and C at different inlet velocities are shown in Figure 35. The results show that monitoring point C exhibits the highest erosion rate. Furthermore, as the inlet velocity increases, the maximum erosion rate of the desander increases, and the slope of the corresponding curve rises significantly. This trend can be attributed to two main factors. First, higher inlet velocity significantly increases the frequency of contact between sand particles and the pipe wall. Second, it raises the relative velocity between the particles and the wall surface, which simultaneously enhances particle kinetic energy and impact pressure. The combined effect of these factors results in a significant increase in erosion rate with rising inlet velocity.

From an engineering application perspective, excessively high inlet velocity exacerbates wall erosion and shortens the sand separator’s service life, while a moderate increase in inlet velocity can improve separation efficiency. Therefore, in practical applications at shale gas well sites, it is essential to balance the relationship between inlet velocity and separation efficiency. This ensures effective gas–solid separation while minimizing wall erosion to the greatest extent possible, thereby achieving long-term stable operation of the sand separator.

#### 4.3.2. Effect of Operating Pressure on Wall Erosion in Cyclone Desanders

This section focuses on the impact of operating pressure on wall erosion in cyclone desanders. The study investigates erosion characteristics under four operating pressure conditions—45 MPa, 40 MPa, 38 MPa, and 35 MPa—while maintaining a fixed inlet velocity of 15 m/s and a constant particle concentration of 0.001 kg/m^3^.

Changes in operating pressure directly alter gas density and viscosity, thereby influencing the internal flow field within the separator. These flow field modifications, in turn, affect particle motion and wall erosion processes. Consequently, investigating the impact of operating pressure on erosion is essential for comprehensively understanding the separator’s operational characteristics under various conditions, providing theoretical support for design optimization and operational parameter control [40].

**Figure 36:**
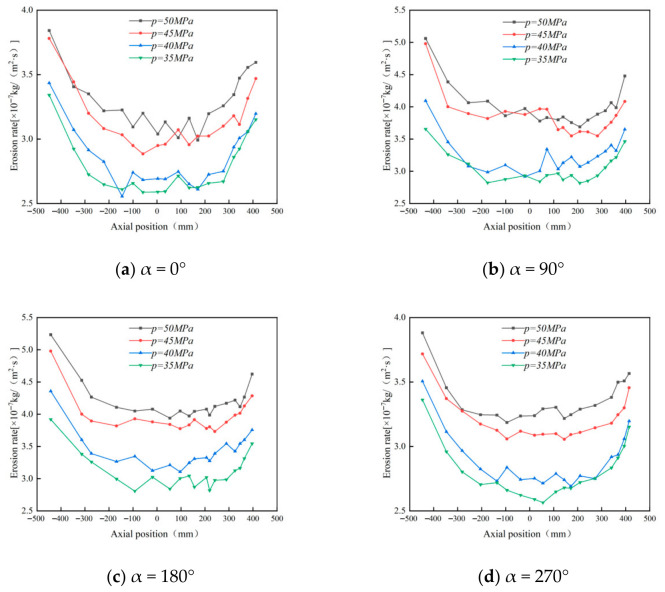
Effect of operating pressure on wall erosion in the cyclone desander.

**Figure 37:**
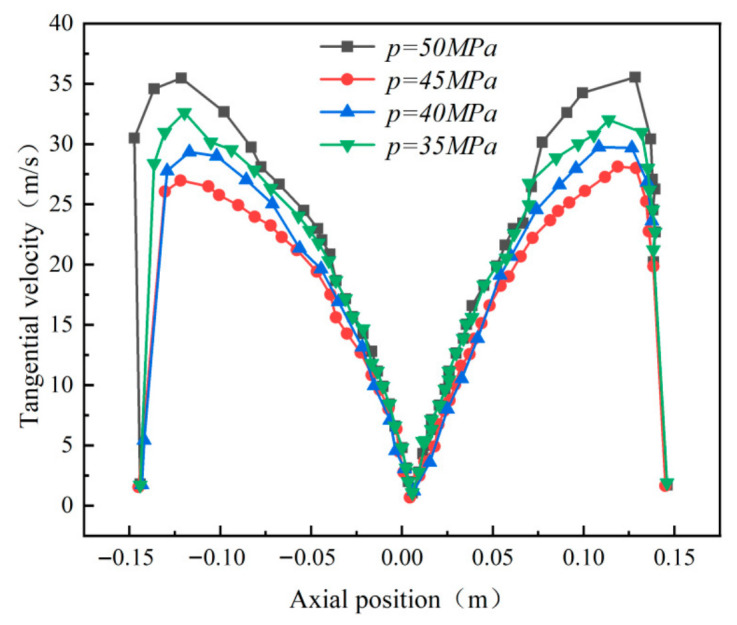
Tangential velocity curves of different inlet aspect ratios at y = 200.

**Figure 38:**
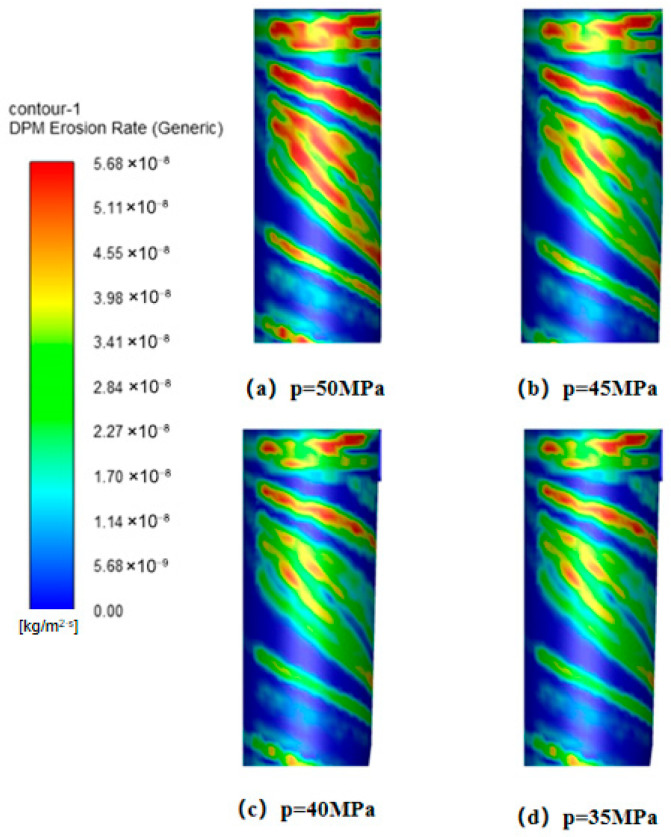
Erosion of the cylinder under different operating pressures.

Simulation results (Figure 36) reveal that peak wall erosion values across all measurement directions are concentrated in the lower conical region. While the overall erosion effect of operating pressure on the cylindrical and conical sections is limited, its impact is particularly pronounced in the inlet region and lower conical section. Specifically, erosion peaks in these two areas exhibit a clear increasing trend with rising operating pressure. Flow field analysis (Figure 37) indicates that at a constant inlet velocity of 15 m/s, the tangential velocity on the cone wall (y = 400 mm) continuously increases with rising operating pressure. This tangential velocity enhancement boosts the gas stream’s particle-carrying capacity, significantly altering particle trajectories and spatial distribution within the desander. This provides a flow-field-level explanation for the observed erosion characteristics. Comparative erosion contour plots (Figure 38) further confirm a positive correlation between operating pressure and wall erosion rate, with erosion being most pronounced at 50 MPa.

It should be noted that while the increased tangential velocity resulting from higher operating pressure effectively enhances the gas–solid separation efficiency of the desander, it simultaneously elevates the erosion rate of particles on the wall surface. Therefore, in engineering applications, an appropriate operating pressure must be selected to balance separation efficiency improvement with wall erosion control, ensuring the long-term stable and efficient operation of the desander.

### 4.4. Synthesis of Erosion Mechanisms and Engineering Implications

Across the single-factor simulations, the erosion distribution was governed by three coupled mechanisms: tangential momentum enhancement, particle–wall collision frequency, and local particle accumulation near geometric transitions. The inlet, conical section, and dust discharge outlet were repeatedly identified as erosion-prone regions, which is consistent with the field monitoring locations selected for model validation. Among the investigated parameters, inlet velocity, inlet aspect ratio, and particle concentration mainly intensified erosion by increasing particle kinetic energy or the number of particle–wall impacts. By contrast, cylinder radius, cone length, dust outlet diameter, and exhaust port diameter primarily modified the internal vortex structure and redistributed particle trajectories. Therefore, although the numerical values of the optimal structural parameters are specific to the M shale-gas wellhead, the dominant erosion mechanisms and the relative importance of key factors provide transferable guidance for similar high-pressure cyclone desanders.

## 5. Parameter Optimization Analysis of Cyclone Desander Based on Response Surface Methodology

The maximum local erosion rate was selected as a conservative damage index to identify the most vulnerable wall region. The purpose of the response surface optimization was not to maximize erosion, but to minimize the peak erosion rate and improve the service life of the cyclone desander.

### 5.1. Significance Analysis of Cyclone Desander Parameters

Based on the simulated effects of structural, medium, and operating parameters on the erosion rate of the cyclone separator, the data were analyzed using SPSSAU software. The erosion rate was set as the dependent variable, with the following as fixed factors: inlet aspect ratio, cylinder diameter, cone diameter, dust discharge port diameter, exhaust port diameter, particle size, particle concentration, inlet velocity, and operating pressure. The ANOVA results are presented in Table 4.

As shown in the table above, factors with *p* < 0.05 are significant factors, including: inlet aspect ratio, cylinder diameter, dust outlet diameter, particle size, particle concentration, and inlet velocity. Factors with *p* < 0.1 are moderately significant factors, including: cone diameter and operating pressure. The exhaust port diameter is a non-significant factor. The influencing factors can be ranked based on their *p*-values:

Inlet velocity > Inlet aspect ratio > Particle concentration > Cylinder diameter > Dust outlet diameter > Particle size > Operating pressure > Cone diameter > Exhaust port diameter.

Ultimately, considering *p*-values (significance) comprehensively, six factors—inlet aspect ratio, cylinder diameter, dust outlet diameter, particle size, particle concentration, and inlet velocity—were selected for subsequent multifactor interaction analysis.

### 5.2. Box–Behnken Response Surface Methodology for Analyzing the Interactive Effects on Erosion Rate

Design-Expert 13 software was employed to perform data fitting for six factors: inlet aspect ratio, cylinder diameter, dust outlet diameter, particle size, particle concentration, and inlet velocity.

#### 5.2.1. Box–Behnken Experiment

Based on prior simulation results, a 6-factor, 3-level response surface analysis experiment was conducted using dust outlet diameter A, particle size B, inlet velocity C, cylinder radius D, inlet aspect ratio E, and particle concentration F, with the erosion rate of the cyclone desander as the response value. The factor levels for the response surface experiment are shown in Table 5, and the simulation results are presented in Table 6.

#### 5.2.2. Regression Equation Fitting and Analysis of Variance

This study analyzed experimental data using Design-Expert 13 software. Based on the response values from 54 experimental groups, regression fitting was performed to construct a mathematical model. The maximum erosion rate ultimately obtained is shown in Equation (12).

(12) $$\begin{array}{l} \begin{array}{l} E=2.29+0.0421A+0.1250B+0.5012C+0.1683D+0.4225E+0.2037F\\ -0.1000AB-0.2538AC-0.2413AD-0.0750AE-0.1337AF\\ -0.0800BC-0.6150BD-0.1200BE-0.2925BF-1.29CD\\ +0.2275CE+0.0444CF+0.0650DE-0.0500DF-0.3975EF \end{array}\\ +0.9815A^{2}+1.05B^{2}+1.01C^{2}+0.5253D^{2}+0.8157E^{2}+1.14F^{2} \end{array}$$

A variance analysis statistical test was conducted on the predictive model for maximum erosion rates. The F-value serves as an indicator measuring the extent to which each independent variable influences the dependent variable; a higher value signifies greater significance of the corresponding variable’s effect. In the significance test, when the probability value P of the initial model is less than 0.05, it indicates that the predictive model possesses strong statistical significance. The established model demonstrated extremely high statistical significance, with an F-value of 50.18 and a *p*-value far below 0.0001 (*p* < 0.0001). This indicates the model possesses exceptionally high statistical significance. The coefficient of determination (R2) reached 0.9812, approaching 1.0, signifying the model exhibits a very high degree of data fit and strong reliability.

## 6. Engineering Implications for Optimized Geometry and Material-Based Erosion Protection

### 6.1. Optimized Geometry and Erosion Reduction Mechanism

**Figure 39:**
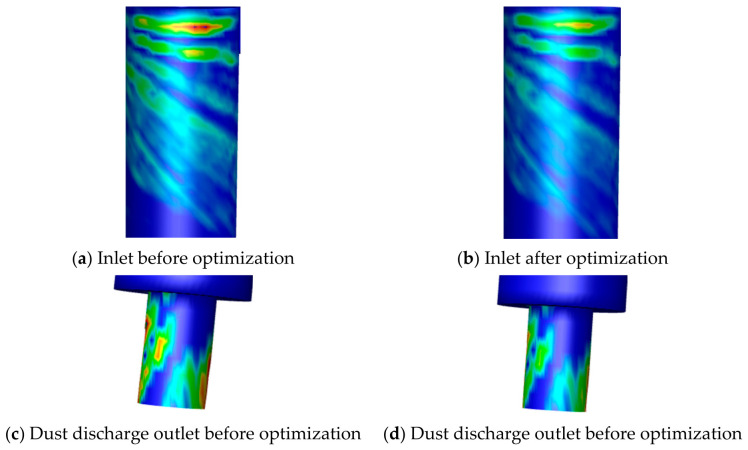
Comparison of erosion before and after cyclone optimization.

Based on the response surface optimization, the optimized structural parameters for reducing the maximum local erosion rate were obtained as follows: inlet aspect ratio of 3, cylinder radius of 75 mm, cone length of 350 mm, dust discharge outlet diameter of 45 mm, and exhaust port diameter of 50 mm. It should be noted that these values represent the multi-factor optimized geometry, whereas the values discussed in the single-factor analysis were candidate values obtained under isolated parameter variation. Compared with the original geometry, the optimized geometry reduced the maximum erosion rate at the inlet and dust discharge outlet by 20.4% and 21.8%, respectively. Therefore, structural optimization of the cyclone desander achieves a remarkable reduction in erosion. Figure 39 shows the erosion rate contours at the inlet and dust outlet of the cyclone desander before and after optimization.

The erosion reduction can be attributed to the improved vortex structure and reduced local particle–wall impact intensity. The optimized inlet aspect ratio helps stabilize the tangential flow entering the desander. The selected cylinder radius and cone length reduce excessive near-wall tangential velocity and particle impact energy in the lower conical region. In addition, the optimized dust discharge outlet diameter decreases particle accumulation and repeated impacts near the dust outlet. These results indicate that geometry optimization can reduce local erosion by regulating the swirling flow field, particle trajectories, and wall-impact behavior.

### 6.2. Material-Based Protection Strategies for Erosion-Prone Regions

The CFD results indicate that the inlet and dust discharge outlet are the most erosion-prone regions of the cyclone desander. Therefore, material-based protection should be preferentially applied to these local high-risk regions rather than uniformly strengthening the entire separator wall. Local wall thickening was proposed for the inlet and dust discharge outlet. Based on the calculated wall-thinning rate, the estimated service life could be extended by approximately 1.15 years.

Material selection is also important because erosion resistance is generally related to hardness, elastic modulus, microstructure, and surface modification. For the erosion-prone regions identified in this study, hard coatings, ceramic-based coatings, cermet coatings, and elastic or energy-absorbing coatings can be considered. Hard and ceramic-based coatings can resist micro-cutting caused by quartz particles, while elastic coatings can absorb part of the particle impact energy and reduce energy transfer to the substrate.

**Figure 40:**
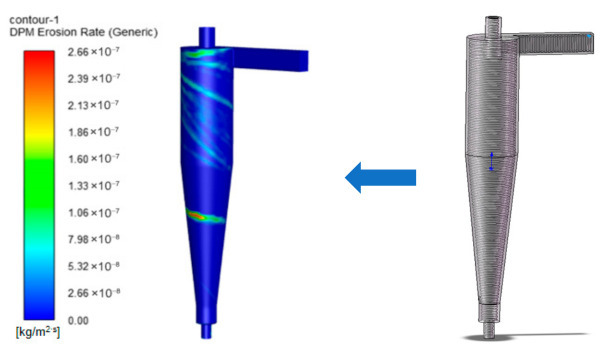
Erosion rate contour and model of desander with bionic ribs.

In addition, a bionic rib structure was evaluated as a surface-protection design. Under the same numerical operating conditions, the maximum erosion rate decreased to 2.23 × 10^−7^ kg/(m^2^·s), corresponding to a 58% reduction compared with the original structure. This indicates that surface-structure modification can further reduce particle–wall impact intensity and improve erosion resistance. However, the coating and bionic-rib protection strategies in this study were evaluated mainly from numerical and engineering perspectives. Further coating erosion tests, material characterization, and field exposure experiments are still required to verify their long-term performance under shale-gas wellhead conditions. The cyclone desander model with convergent rib designs is shown in Figure 40.

## 7. Conclusions

In this study, a cyclone desander used at the M shale-gas wellhead was investigated through field measurements, CFD simulation, significance analysis, and response surface methodology. The main conclusions are as follows:

(1) A CFD-based erosion model of the cyclone desander was established and validated using field wall-thickness monitoring data. The relative simulation errors at monitoring points A, B, and C were 4.73%, 4.45%, and 2.06%, respectively, with an average relative error of 3.75%, indicating that the model can reasonably predict the main erosion-prone regions of the desander.
(2) The effects of structural parameters, particle properties, and operating conditions on wall erosion were systematically analyzed. The results showed that inlet aspect ratio, cylinder radius, cone length, dust discharge outlet diameter, particle size, particle concentration, inlet velocity, and operating pressure all affected the erosion intensity, while the inlet and dust discharge outlet remained the most vulnerable regions.
(3) Significance analysis identified six key factors influencing the maximum erosion rate: inlet aspect ratio, cylinder radius, dust discharge outlet diameter, particle size, particle concentration, and inlet velocity. Based on the response surface optimization, the optimized structural parameters were determined as an inlet aspect ratio of 3, a cylinder radius of 75 mm, a cone length of 350 mm, a dust discharge outlet diameter of 45 mm, and an exhaust port diameter of 50 mm.
(4) Compared with the original geometry, the optimized desander reduced the maximum erosion rate from 5.34 × 10^−7^ to 4.25 × 10^−7^ kg/(m^2^·s) at the inlet and from 5.22 × 10^−7^ to 4.08 × 10^−7^ kg/(m^2^·s) at the dust discharge outlet, corresponding to reductions of 20.4% and 21.8%, respectively.
(5) Local wall thickening at the inlet and dust discharge outlet was found to be beneficial for service-life improvement. Based on the calculated wall-thinning rate, the estimated service life of the desander could be extended by approximately 1.15 years.
(6) A bionic-rib protection design was further evaluated. Under the same numerical operating conditions, the maximum erosion rate decreased to 2.23 × 10^−7^ kg/(m^2^·s), corresponding to a 58% reduction compared with the original structure. This indicates that combining geometry optimization with local structural protection is a promising strategy for improving the erosion resistance and service reliability of shale-gas wellhead cyclone desanders.

## Figures and Tables

**Table 1:** Structural Parameters.

Structure Name	Dimension (mm)	Structure Name	Dimension (mm)
Column Segment Diameter	150	Small Column Segment Diameter	60
Column Segment Length	350	Small Column Segment Length	55
Exhaust Port Diameter	45	Pipe Segment Height	450
Insertion Depth	60	Dust Port Diameter	30
Inlet Dimensions	(60, 38.82)		

**Table 2:** Gas composition.

Component Name	Mole Fraction (%)
C_1_	98.02
C_2_	0.2202
N_2_	1.759

**Table 3:** Different Aspect Ratio Structural Parameters.

Entrance Aspect Ratio (a/b)	a	b	Hydraulic Diameter
1	48 mm	48 mm	48
1.5	60 mm	38.82 mm	47
2.5	76 mm	30 mm	43
5	105 mm	21 mm	35

**Table 4:** ANOVA results.

Factor	Sum of Squares	Degrees of Freedom	Mean Square	F	*p*
Inlet velocity	100.00	2	50.00	25.00	0.001
Entrance Aspect Ratio	50.00	2	25.00	12.50	0.002
Particle Concentration	30.00	2	15.00	7.50	0.005
Cylinder Diameter	20.00	2	10.00	5.00	0.010
Dust Outlet Diameter	15.00	2	7.50	3.75	0.020
Particle Size	10.00	2	5.00	2.50	0.050
Operating Pressure	8.00	2	4.00	2.00	0.060
Cone Diameter	5.00	2	2.50	1.25	0.100
Exhaust Port Diameter	0.00	2	0.00	0.00	1.000

**Table 5:** Experimental factors and levels.

Factor	Factor Name	Factor Unit	Level 1	Level 2	Level 3
A	Dust Outlet Diameter	mm	30	45	60
B	Particle Size	μm	5	7.5	10
C	Inlet velocity	m/s	5	15	25
D	Cylinder Radius	mm	60	75	90
E	Entrance Aspect Ratio	—	1	3	5
F	Particle Concentration	Kg/s	0.0002	0.0007	0.0012

**Table 6:** Randomized experimental groups and simulation results.

Serial Number	Factor: A	Factor: B	Factor: C	Factor: D	Factor: E	Factor: F	Response Value
	Dust Outlet Diameter (mm)	Particle Size (μm)	Inlet Velocity (m/s)	Cylinder Radius (mm)	Entrance Aspect Ratio	Particle Concentration (kg/s)	Erosion Rate 10^−7^ kg/ (m^2^·s)
1	45	7.5	25	60	3	0.0012	6.99
2	45	7.5	15	75	3	0.0007	2.12
3	30	7.5	15	90	5	0.0007	5.43
4	60	7.5	15	90	1	0.0007	4.39
5	45	7.5	25	60	3	0.0002	6.29
6	30	7.5	5	75	3	0.0002	4.28
7	45	10	25	75	1	0.0007	5.43
8	45	7.5	15	75	3	0.0007	2.15
9	60	5	15	90	3	0.0007	5.49
10	45	10	25	75	5	0.0007	6.41
11	30	10	15	90	3	0.0007	4.95
12	30	7.5	15	60	1	0.0007	3.89
13	30	10	15	60	3	0.0007	4.99
14	45	5	25	75	5	0.0007	6.18
15	45	7.5	5	60	3	0.0002	2.81
16	30	7.5	25	75	3	0.0012	6.73
17	45	5	15	75	1	0.0012	5.39
18	60	7.5	15	90	5	0.0007	5
19	30	7.5	25	75	3	0.0002	5.48
20	45	7.5	15	75	3	0.0007	2.31
21	60	5	15	60	3	0.0007	4.37
22	45	7.5	5	90	3	0.0012	6.04
23	30	7.5	15	90	1	0.0007	4.28
24	45	5	15	75	5	0.0012	5.85
25	60	10	15	90	3	0.0007	3.92
26	45	5	15	75	1	0.0002	4.01
27	30	5	15	90	3	0.0007	5.86
28	60	10	15	60	3	0.0007	5.52
29	45	10	15	75	1	0.0002	4.76
30	45	5	15	75	5	0.0002	5.86
31	30	5	15	60	3	0.0007	3.7
32	45	7.5	15	75	3	0.0007	2.45
33	45	5	5	75	1	0.0007	3.96
34	60	7.5	5	75	3	0.0002	4.99
35	30	7.5	5	75	3	0.0012	4.9
36	60	7.5	15	60	1	0.0007	4.39
37	60	7.5	15	60	5	0.0007	4.98
38	45	7.5	15	75	3	0.0007	2.37
39	60	7.5	25	75	3	0.0002	5.6
40	45	10	15	75	5	0.0012	4.99
41	45	5	25	75	1	0.0007	4.55
42	60	7.5	5	75	3	0.0012	5.5
43	60	7.5	25	75	3	0.0012	5.89
44	45	7.5	25	90	3	0.0012	4.52
45	45	10	5	75	5	0.0007	5.23
46	45	7.5	15	75	3	0.0007	2.34
47	45	5	5	75	5	0.0007	4.55
48	45	10	15	75	5	0.0002	6.37
49	45	7.5	5	60	3	0.0012	3.22
50	45	10	5	75	1	0.0007	5.03
51	30	7.5	15	60	5	0.0007	4.54
52	45	7.5	5	90	3	0.0002	5.69
53	45	10	15	75	1	0.0012	5.17
54	45	7.5	25	90	3	0.0002	4.16

## Data Availability

The original contributions presented in this study are included in the article. Further inquiries can be directed to the corresponding author.
